# 3D Printing of a Vascularized Mini‐Liver Based on the Size‐Dependent Functional Enhancements of Cell Spheroids for Rescue of Liver Failure

**DOI:** 10.1002/advs.202309899

**Published:** 2024-02-21

**Authors:** Jiabin Zhang, Xiaodie Chen, Yurong Chai, Chenya Zhuo, Yanteng Xu, Tiantian Xue, Dan Shao, Yu Tao, Mingqiang Li

**Affiliations:** ^1^ Laboratory of Biomaterials and Translational Medicine Center for Nanomedicine The Third Affiliated Hospital, Sun Yat‐Sen University Guangzhou 510630 China; ^2^ Guangdong Provincial Key Laboratory of Liver Disease Guangzhou 510630 China; ^3^ Institute of Life Sciences School of Medicine South China University of Technology Guangzhou 510006 China

**Keywords:** 3D printing, hepatic lobules, liver failure, mesenchymal stromal/stem cells, vasculatures

## Abstract

The emerging stem cell‐derived hepatocyte‐like cells (HLCs) are the alternative cell sources of hepatocytes for treatment of highly lethal acute liver failure (ALF). However, the hostile local environment and the immature cell differentiation may compromise their therapeutic efficacy. To this end, human adipose‐derived mesenchymal stromal/stem cells (hASCs) are engineered into different‐sized multicellular spheroids and co‐cultured with 3D coaxially and hexagonally patterned human umbilical vein endothelial cells (HUVECs) in a liver lobule‐like manner to enhance their hepatic differentiation efficiency. It is found that small‐sized hASC spheroids, with a diameter of ≈50 µm, show superior pro‐angiogenic effects and hepatic differentiation compared to the other counterparts. The size‐dependent functional enhancements are mediated by the Wnt signaling pathway. Meanwhile, co‐culture of hASCs with HUVECs, at a HUVECs/hASCs seeding density ratio of 2:1, distinctly promotes hepatic differentiation and vascularization both in vitro and in vivo, especially when endothelial cells are patterned into hollow hexagons. After subcutaneous implantation, the mini‐liver, consisting of HLC spheroids and 3D‐printed interconnected vasculatures, can effectively improve liver regeneration in two ALF animal models through amelioration of local oxidative stress and inflammation, reduction of liver necrosis, as well as increase of cell proliferation, thereby showing great promise for clinical translation.

## Graphical abstract

Based on the size‐dependent functional enhancements of human mesenchymal stromal/stem cell spheroids and their interaction with endothelial cells during cell co‐culture and hepatic differentiation, a vascularized mini‐liver, consisting of hexagonally patterned vasculatures and hepatocyte‐like cell spheroids, is fabricated by 3D coaxial bioprinting and ectopically implanted for the effective alleviation of acute liver failure.

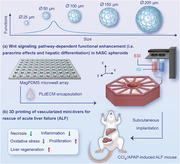

## 1 Introduction

Liver is a vital organ responsible for a variety of functions, including glycogen and lipid storage, drug detoxification, as well as secretion of serum proteins and bile. The basic unit of liver is the hexagonal lobule, in which blood flows from the peripheral hepatic veins and arteries toward the central vein between hepatocytes through sinusoids.^[^ ^1^ ^]^ Owing to the essential roles of liver in the maintenance of homeostasis, the lethal acute liver failure (ALF) requires timely medical interventions.^[^ ^2^ ^]^ Basically, orthotopic liver transplantation is the gold standard treatment for end‐stage ALF, however, the available liver donors are far from the clinical demands.^[^ ^2b^ ^]^

Although hepatocyte therapy has shown some clinical successes in the treatment of ALF, there are still barriers compromising their therapeutic efficacy, including cell shortage and harsh host environment.^[^ ^2a^ ^]^ Due to the unrealistic isolation of autologous hepatocytes from ALF patients as well as the difficulty of allogenic cell expansion and function maintenance in vitro, the emerging stem cell‐derived hepatocyte‐like cells (HLCs) have gained great interest as the alternative cell sources for ALF treatment.^[^ ^3^ ^]^ A number of studies have shown successful hepatic differentiation of embryonic stem cells (ESCs), induced pluripotent stem cells (iPSCs), and mesenchymal stromal/stem cells (MSCs) by sequential cell culture in different inductive cocktails.^[^ ^4^ ^]^ MSCs are particularly of interest due to their convenient access, low risk of tumorigenicity, and lack of ethical issues.^[^ ^5^ ^]^ However, the therapeutic efficacy of stem cell‐derived HLCs was often less effective than the mature hepatocytes due to their immature differentiation.^[^ ^6^ ^]^ Recent studies have shown that engineering pluripotent stem cells (iPSCs and ESCs) into multicellular spheroids could enhance their hepatic differentiation efficiency.^[^ ^4a,c^ ^]^ Nevertheless, most studies only considered the mass and gas penetrating limit distance (≈200 µm) of cell spheroids, while ignoring their size‐dependent functional differences.^[^ ^7^ ^]^ Alternatively, co‐culture of stem cell‐derived hepatocyte‐like cells with endothelial cells also contributed to their maturation, especially when endothelial cells were hexagonally patterned.^[^ ^4^, ^8^ ^]^ However, the simple structures fail to mimic the complex, interconnected hepatic vasculatures found in vivo.^[^ ^1^ ^]^ In addition, there is limited research on directly inducing hepatic differentiation of MSCs during their co‐culture with endothelial cells.^[^ ^9^ ^]^

**Figure 1:**
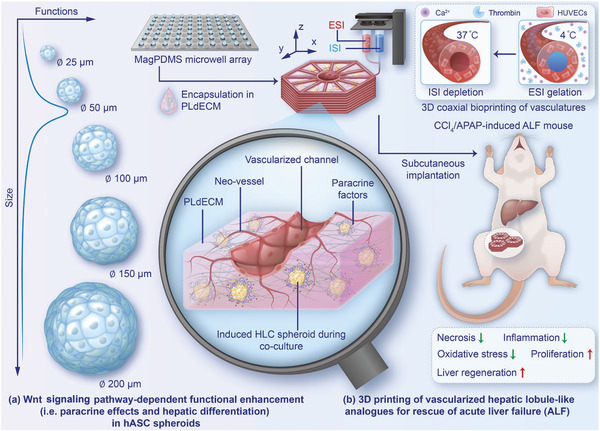
A schematic illustrating 3D coaxial bioprinting of vascularized hepatic lobule‐like analogs using functional human mesenchymal stromal/stem cell (hASC) spheroids for the rescue of acute liver failure (ALF). a) Wnt signaling pathway‐dependent functional enhancement in hASC spheroids, including paracrine effects and hepatic differentiation. b) The hexagonal vasculatures are first constructed by 3D coaxial bioprinting of endothelial cell‐incorporated external supporting ink (ESI) and internal sacrificing ink (ISI). The hollow channels are created by depletion of ISI after ESI crosslinking. The stuffing parenchyma ink (SPI) containing functional hASC spheroids is subsequently added to fulfill the radial intervals. During cell co‐culture, the hASC spheroids are differentiated into hepatocyte‐like cell (HLC) spheroids. The modular vascularized hepatic lobule analogs can be subcutaneously implanted near the inguinal fat pads that are rich in blood vessels for effective and efficient ALF treatment.

In this regard, we fabricated a 3D‐printed vascularized mini‐liver using functional human adipose‐derived mesenchymal stromal/stem cell (hASC) spheroids for the treatment of ALF in the current study (**Figure** **1**). Within the gas/mass penetrating limit distance, we found that small‐sized hASC spheroids (S spheroid), at an approximate diameter of 50 µm, showed superior pro‐angiogenic effects and hepatic differentiation efficiency compared to the equivalent number of dissociated single cells and other varying‐sized hASC spheroids (XS (≈25 µm), M (≈100 µm), L (≈150 µm), XL (≈200 µm)). We demonstrated that the Wnt signaling pathway was implicated in the size‐dependent functional enhancements. In particular, the vascularization and hepatic differentiation could be further improved by co‐culture of hASCs with 3D coaxially and hexagonally patterned human umbilical vein endothelial cells (HUVECs) at a HUVECs/hASCs cell seeding density ratio of 2:1. The subcutaneously implanted mini‐liver, consisting of HLC spheroids and 3D‐printed interconnected vasculatures, could effectively alleviate oxidative stress and inflammation, decrease tissue necrosis, and promote cell proliferation in the host livers of carbon tetrachloride (CCl_4_)‐ or acetaminophen (APAP)‐induced ALF mice, thereby showing great promise for clinical translation.

## 2 Results

### 2.1 3D Coaxial Printing of Hexagonal Liver Lobule‐Like Vasculatures

**Figure 2:**
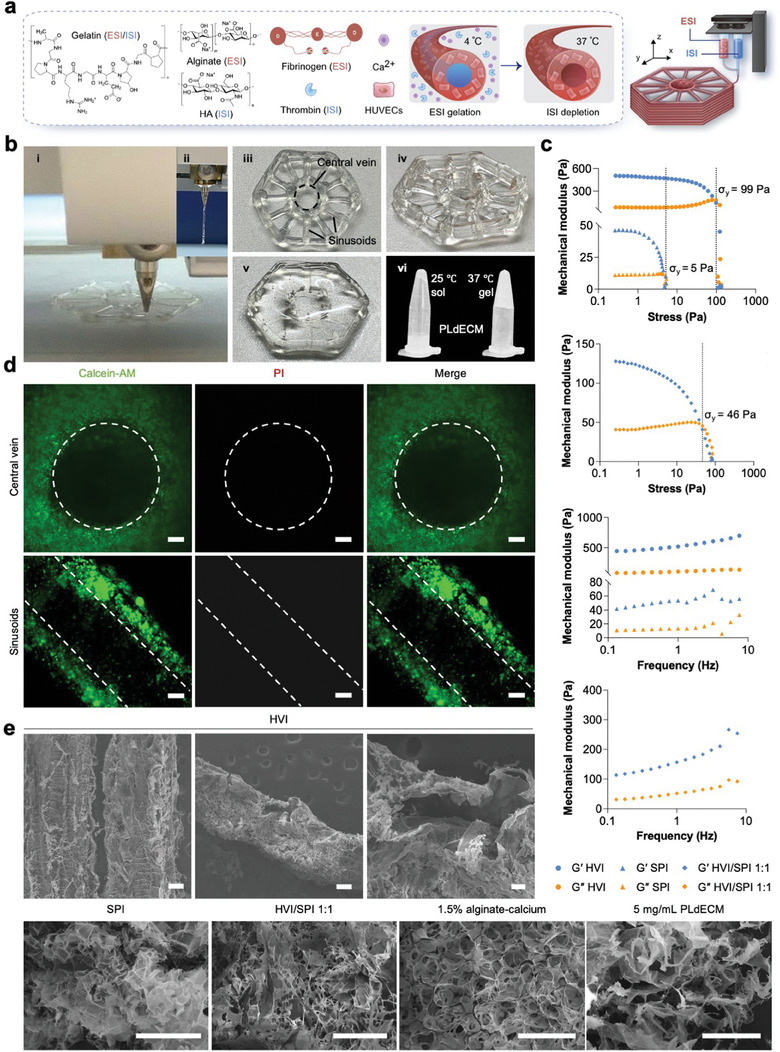
Characterization of vascularized hepatic lobule‐like analogs. a) A schematic image illustrates the procedures of 3D coaxial bioprinting. b) 3D coaxial printing of a hepatic lobule‐like structure using (i‐v) hexagonal vasculature ink (HVI, 1.5% alginate‐calcium + 1 mg mL^−1^ fibrin) and (vi) stuffing parenchyma ink (SPI, 10 mg mL^−1^ porcine liver‐derived decellularized extracellular matrix (PLdECM)). c) Rheology study of different inks using stress sweep mode (frequency = 0.1 Hz) or frequency sweep mode (stress = 0.1 Pa). G′: storage modulus; G″: loss modulus. d) Live & dead staining of the 3D‐printed HUVECs on day 1. Green: live cells; red: dead cells. Scale bar: 400 µm (top) and 200 µm (bottom). e) SEM images of 3D‐printed HVI into hollow channels (two adjacent channels (left) and partially disrupted single channel at low (middle) and high (right) magnifications) and different hydrogel inks. Scale bar: 200 µm.

Inspired by the specific architecture of liver lobules, here we biofabricated a similar hexagonal structure with interconnected vasculatures, including a central vein and radial sinusoids by 3D coaxial bioprinting (**Figure** **2**a). It was shown that a straight and consecutive strand could be extruded during 3D coaxial printing, indicating the printability of the formulating internal sacrificing ink (ISI) and external supporting ink (ESI) (Figure 2bi‐ii). During 3D coaxial printing, the released thrombin from the central ISI would transform the surrounding fibrinogen component of ESI into fibrin and initiate crosslinking, and herein contributed to the stability of the 3D‐printed hexagonal skeleton together with the low temperature gelation property of gelatin (Figure 2biii‐iv). To improve the mechanical strength of the structure, a further mild crosslinking of the alginate and fibrinogen components of ESI in the calcium/thrombin solution was performed (Figure 2bv). After depletion of ISI in warm 1× PBS, the remaining crosslinked hexagonal vasculature ink (HVI) could form interconnected hollow channels. Due to the good maintenance of bioactive naïve liver ingredients, a thermosensitive porcine liver‐derived decellularized extracellular matrix (PLdECM) hydrogel was cast into the intervals of radial sinusoids as the stuffing parenchyma ink (SPI) for subsequent 3D stem cell culture and differentiation (Figure 2bvi).^[^ ^4g^ ^]^ The addition of HVI distinctly increased the yield stress σ_y_ of SPI from 5 to 46 Pa (Figure 2c). During the frequency sweep from 0.1 to 10 Hz, the crosslinked HVI, SPI as well as their hybrid, showed stable gel status (Figure 2c). The degradation study revealed that both SPI and HVI were biodegradable, with SPI exhibiting a faster degradation rate compared to HVI (Figure S1a,b, Supporting Information). When prepared in bulk, the HVI showed a slower release profile of rhodamine B than SPI (Figure S1c, Supporting Information). Notably, only 1.19% of rhodamine B was released from the bulk HVI within 30 min. In contrast, a distinct perfusion of rhodamine B was observed in the 3D‐printed hollow hexagons, as evidenced in both Figures S1c,S2 (Supporting Information). Although the pro‐angiogenic potentials of SPI and HVI were not comparable to that of Matrigel, SPI supported persisting endothelial cell proliferation (Figure S3, Supporting Information). After 3D coaxial bioprinting of HUVECs into hexagonal interconnected vasculatures, the cells showed good viability, revealing good biocompatibility of the formulating inks and the 3D printing processes (Figure 2d). The hollow channels of the hexagonal vasculatures were also evidenced by SEM in addition to the porous crosslinked inks (Figure 2e).

### 2.2 Size‐Dependent Functional Enhancements of hASC Spheroids

**Figure 3:**
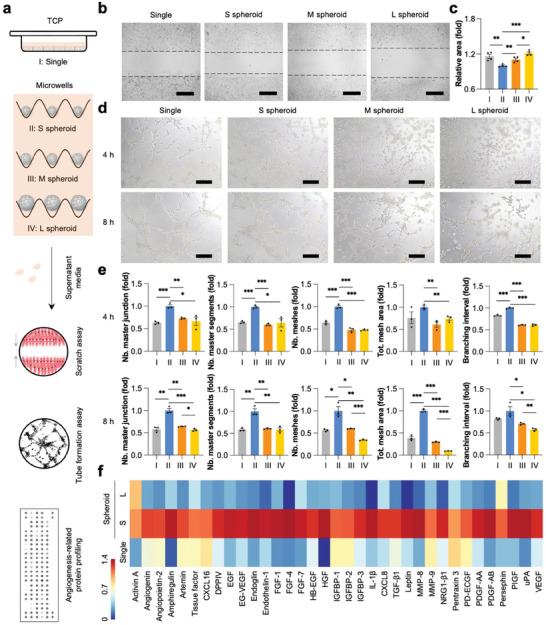
Pro‐angiogenic effects of hASC spheroids. a) A schematic image illustrates the pro‐angiogenic effects of hASC spheroids/single cells‐derived secretomes. b) Optical microscopy images of HUVEC migration at 36 h. Scale bar: 500 µm. c) Quantification of the remaining area. All data are normalized to “S spheroid” group and presented as mean ± SEM, *n* = 4. d) Tube formation of HUVECs cultured with different hASC supernatant media at 4 and 8 h. Scale bar: 200 µm. e) Quantification of the formed tubes. All data are normalized to the value of “S spheroid” group and presented as mean ± SEM, *n* = 3. (f) Angiogenesis‐related proteins in the supernatant media of single cells (Single), small‐sized spheroids (S spheroid), and large‐sized spheroids (L spheroid) characterized by human angiogenesis array. Each component was measured in duplicate. All values were normalized to the initial cell number. 0.01 < *p < 0.05, 0.001 < **p < 0.01, and ***p < 0.001.

**Figure 4:**
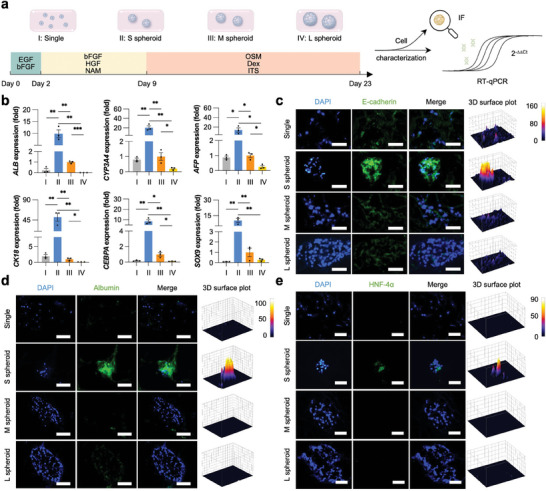
Hepatic differentiation of hASCs. a) A schematic illustrates the hepatic differentiation of hASCs cultured as single cells (Single) or different‐sized cell spheroids (S spheroid, M spheroid, L spheroid) in PLdECM hydrogels. b) The relative mRNA expression of hepatic genes in hASCs cultured inside PLdECM hydrogels as single cells or cell spheroids with distinct sizes. All data are normalized to the value of “M spheroid” group and presented as mean ± SEM, *n* = 3. c–e) Immunofluorescent staining of E‐cadherin, albumin, and HNF‐4α in hepatic differentiation‐induced hASCs either as single cells or cell spheroids with distinct size in PLdECM hydrogels. Green: Alexa Fluor 488‐labeled E‐cadherin, albumin, or HNF‐4α; Blue: DAPI‐labeled cell nuclei. Scale bar: 50 µm. 0.01 < *p < 0.05, 0.001 < **p < 0.01, and ***p < 0.001.

To investigate the functional difference of hASC spheroids at varying diameters within the maximal penetrating distance of oxygen and nutrition (≈200 µm), three different‐sized hASC spheroids (50, 100, and 150 µm), in the absence of necrotic core, were generated by magnetic PDMS microwell arrays according to our previous study (Figures S4a–c,S5, Supporting Information).^[^ ^7^, ^10^ ^]^ Compared to dissociated single cells (Single), cell spheroids tended to express a higher level of integrin‐ (*ITGA2* and *ITGB3*), catenin‐ (*CTNNB1*), matrix metalloproteinases‐ (*MMP14* and *MMP3*), paracrine factor‐ (*WLS* and *TGFB3*) related genes but a lower level of the cytoskeleton‐ (*ATCB*), cell proliferation‐ (*PCNA*), and hypoxia‐ (*HIF1A*) related genes (Figure S4d, Supporting Information). Moreover, the unique differences highly depended on the size of hASC spheroids, with superior expression of these genes in S spheroid compared to other counterparts (Figure S4d, Supporting Information). The supernatant media of different‐sized hASC spheroids were harvested to investigate their pro‐angiogenic potentials (**Figure** **3**a). Although the number of cells in the S spheroid was the fewest among all groups, HUVECs incubated with the S spheroid‐derived supernatant medium showed significantly enhanced cell migration in the scratch assay (Figure 3b,c) and tube formation on Matrigel (Figure 3d,e). Moreover, HUVECs showed improved mRNA expression of the angiogenesis‐ (*ANGPT1*), cell proliferation‐ (*MKI67*), and cell migration‐ (*ID1*) related genes when cultured in S spheroid‐derived supernatants (Figure S6, Supporting Information). The tendency was also evidenced in the mRNA expression of paracrine factor‐related genes, such as *FGF2*, *WLS*, and *TGFB1* (Figure S6, Supporting Information). Notably, the receptor genes of stromal cell‐derived factor (SDF‐1), including C‐X‐C motif chemokine receptor 4 (*CXCR4*) and C‐X‐C motif chemokine receptor 7 (*CXCR7*), were downregulated in the HUVECs treated with S spheroid‐derived supernatant media, while the mRNA expression of *MET*, a hepatocyte growth factor (HGF) receptor, was increased, indicating the potential engagement of HGF in the pro‐angiogenic effects (Figure S6, Supporting Information).^[^ ^11^ ^]^ This was further evidenced by a human angiogenesis profiling array that showed superior expression of 35 pro‐angiogenic proteins in the “S spheroid” group (Figure 3f). In addition to the paracrine effects, hASC spheroids, with different diameters, were also induced hepatic differentiation in PLdECM hydrogels using a series of inductive cocktails (**Figure** **4**a). The mRNA expression of hepatocyte‐related genes (*ALB*, *CYP3A4*, *AFP*, *CK18*, *CEBPA*, and *SOX9*) was distinctly upregulated in the “S spheroid” group compared to the “Single” group (Figure 4b). Meanwhile, the functional enhancement highly depended on the size of cell spheroids. Immunofluorescent staining of E‐cadherin, albumin, and HNF‐4α further confirmed the differently expressed proteins (Figure 4c–e). Even after weeks of culture and induced differentiation in the PLdECM hydrogels, hASC spheroids continued to maintain their multicellular morphology (Figure S7, Supporting Information).

We further investigated the size‐dependent functional enhancements of hASC spheroids in a wider diameter range without exceeding the maximal distance (≈200 µm) for sufficient penetration of oxygen and nutrients (Figure S8a, Supporting Information). After the equivalent number of hASCs were seeded on the different‐sized PDMS microwells overnight, multicellular spheroids were formed with distinct sizes (extra‐small spheroid (XS spheroid, 25 µm), small spheroid (50 µm), and extra‐large spheroid (XL spheroid, 200 µm)) (Figures S8b,c,S9, Supporting Information). The genes encoding the cytoskeleton (*ACTB*), integrin (*ITGB3*, *ITGA2*, and *ITGAV*), catenin (*CTNNB1*), matrix metalloproteinases (*MMP14* and *MMP3*), paracrine factors (*VEGFA*, *TGFB3*, *IGF*, and *HGF*), and relevant mediators (*WLS* and *HIF1A*) were significantly upregulated in S spheroid compared to the other counterparts (Figure S8d, Supporting Information). Moreover, the promoted cell migration, proliferation, and angiogenesis were also evidenced in HUVECs treated by S spheroid‐derived supernatant media (Figures S10,S11, Supporting Information). In addition to the differently expressed *ANGPT1* and *CXCR4* genes, the paracrine effect‐related genes, such as *HGF* and *WLS*, were significantly up‐regulated in the HUVECs when cultured by S spheroid‐derived supernatants (Figure S12, Supporting Information). After induced hepatic differentiation in the PLdECM hydrogels, S spheroid still maintained multicellular architecture and showed superior expression of hepatocyte‐related genes at mRNA (*ALB*, *CEBPA*, *AFP*, and *CYP3A4*) and protein (HNF‐4α) levels than the other counterparts (Figures S13,S14, Supporting Information).

### 2.3 Transcriptomics Revealed the Underlying Mechanism for the Size‐Dependent Functional Enhancements in hASC Spheroids

**Figure 5:**
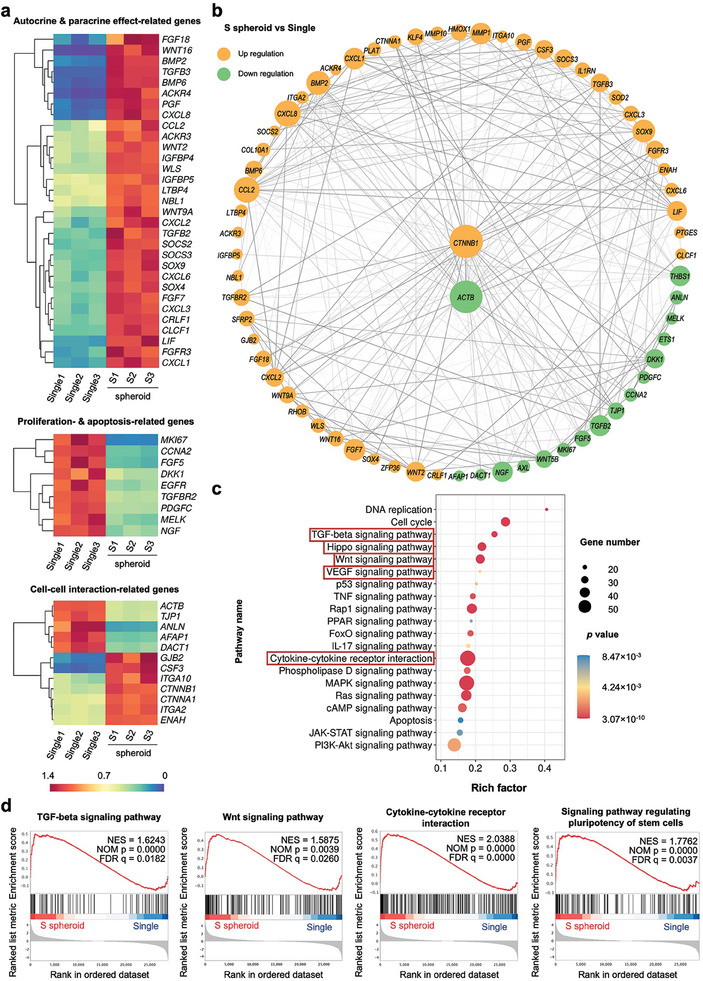
Transcriptome analysis of hASCs either cultured as small‐sized cell spheroids (S spheroid) or dissociated single cells (Single). a) Heat maps of autocrine/paracrine effect‐related genes, proliferation/apoptosis‐related genes, and cell‐cell interaction‐related genes show distinct expression in the hASCs. b) Interaction networks of differently expressed genes. Circle size indicates the interactive frequency and line thickness reflects the strength of data support. c) KEGG analysis reveals the involved signaling pathways for the differently expressed genes between cell spheroids and single cells. d) GSEA plots reveal the potential signaling pathways involved in the functional enhancement of cell spheroids based on the KEGG gene set. *n* = 3, *p* < 0.05.

Further transcriptome sequencing analysis showed that the genes responsible for autocrine/paracrine effects as well as cell‐cell interaction were significantly up‐regulated in the small‐sized hASC spheroids (S1‐3 spheroid) compared with the dissociated single hASCs (Single1‐3), while the mRNA expression of cell proliferation‐ and apoptosis‐related genes was decreased (**Figure** **5**a). Moreover, the gene interaction network showed that the differentially expressed genes were highly involved with *CTNNB1* and *ACTB* genes, indicating the key roles of β‐catenin and β‐actin in the cell signaling processes (Figure 5b). The gene ontology (GO) analysis provided brief information on the changes in biological processes (activated multicellular organism development and cell differentiation but suppressed cell proliferation), cellular components (reduced cytoskeleton) as well as molecular functions (enhanced transcription factor binding) in the S spheroid (Figure S15, Supporting Information). Compared to the dissociated single hASCs, the functional enhancements of cell spheroids were attributed to multiple signaling pathways, such as TGF‐β signaling pathway, Hippo signaling pathway, VEGF signaling pathway, cytokine–cytokine receptor interaction, among which Wnt signaling pathway is particularly of interest due to its important role in the transduction of extracellular signals into cells and initiation of subsequent signaling cascades (Figure 5c). The gene set enrichment analysis (GSEA) showed that the representative Kyoto encyclopedia of genes and genomes (KEGG) signaling pathways, including the TGF‐β signaling pathway, Wnt signaling pathway, cytokine–cytokine receptor interaction, and signaling pathway regulating pluripotency of stem cells, were all upregulated in S spheroid (Figure 5d).

**Figure 6:**
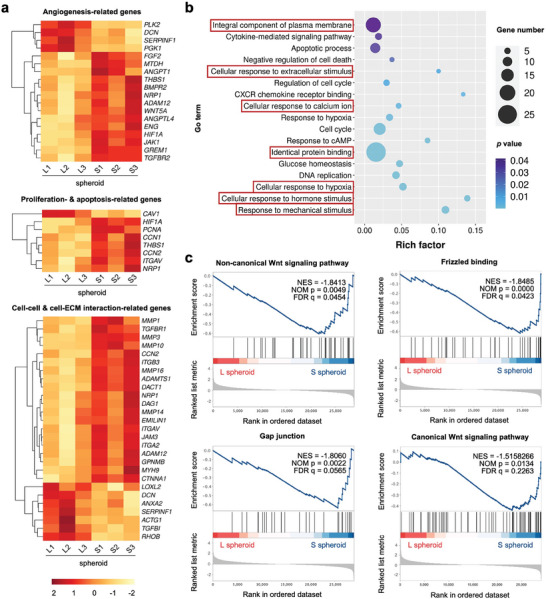
Transcriptome analysis of different‐sized hASC spheroids (S spheroid and L spheroid). a) Heat maps of angiogenesis‐related genes, proliferation/apoptosis‐related genes, and cell‐cell/ECM interaction‐related genes show distinct expression in the different‐sized hASC spheroids. b) GO analysis of the significantly expressed genes in the different‐sized hASC spheroids. c) GSEA plots reveal the potential signaling pathways involved in the size‐dependent functional enhancement of cell spheroids based on the GO gene set. *n* = 3, *p* < 0.05.

The transcriptomics analysis indicated that the angiogenesis‐, cell proliferation‐ and apoptosis‐, as well as cell–cell and cell–ECM interaction‐related genes were differently expressed in the small‐sized hASC spheroids (S1‐3 spheroid) compared to that in the large‐sized cell spheroids (L1‐3 spheroid) (**Figure** **6**a). In particular, *WNT5A*, *WNT5B*, *WNT9A*, *WNT16*, and *CTNNB1* genes were significantly up‐regulated in the S spheroid (Figure S16, Supporting Information). Moreover, the GO analysis revealed the involvement of integral components of the plasma membrane in response to diverse external stimuli (Figure 6b). Accordingly, the GSEA showed that the potential signaling pathways involved in the size‐dependent functional enhancements of hASC spheroids, including the calcium‐dependent non‐canonical Wnt signaling pathway, frizzled binding, gap junction, and canonical Wnt signaling pathway, were all upregulated in S spheroid (Figure 6c).

**Figure 7:**
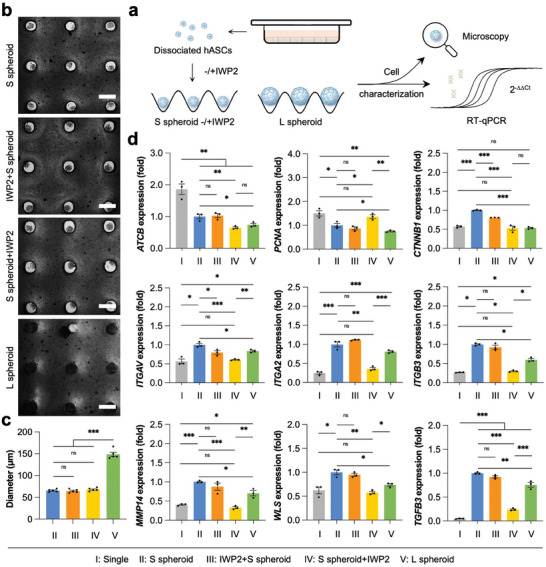
Modulation of Wnt signaling pathway in the size‐dependent functional enhancements of hASC spheroids. a) A schematic illustrates the formation of hASC spheroids without/with the addition of a Wnt signaling inhibitor, IWP2, before or after formation of hASC spheroids. b) Microscopy images of various hASC spheroids with different treatments. Scale bar: 200 µm. c) Quantification of hASC spheroids. Data are presented as mean ± SEM, *n* = 5. d) The relative mRNA expression of *ACTB*, *MKI67*, *PCNA*, *CTNNB1*, *ITGAV*, *ITGA2*, *ITGB3*, *MMP14*, and *TGFB3* genes in hASC spheroids. Data are normalized to the value of “S spheroid” group and presented as mean ± SEM, *n* = 3. Single: single hASCs on 2D TCP without the addition of Wnt inhibitor; S spheroid: small‐sized hASC spheroids without the addition of Wnt inhibitor; IWP2+S spheroid: small‐sized hASC spheroids with the addition of IWP2 before cell aggregation; S spheroid+IWP2: small‐sized hASC spheroids with the addition of IWP2 after cell aggregation; L spheroid: large‐sized hASC spheroids without the addition of Wnt inhibitor. 0.01 < *p < 0.05, 0.001 < **p < 0.01, and ***p < 0.001, not significant (ns) *p* > 0.05.

To investigate the influence of the Wnt signaling pathway on the size‐dependent functional enhancements of hASC spheroids, IWP2 was added into the culture medium to inhibit Wnt production (**Figure** **7**a). Although cell spheroids were still formed overnight and S spheroid size kept consistent regardless of IWP2 treatment (Figure 7b,c), the gene expression profiles showed distinct changes when cells were treated with the inhibitor (Figure 7d). However, only partial genes (*CTNNB1* and *ITGAV*) were slightly downregulated when IWP2 was added before the formation of hASC spheroids (IWP2+S spheroid), indicating that Wnt signaling pathway had minor impacts on cell aggregation and the result was in accordance with the transcriptomic KEGG analysis (Figures 7d and 5c). Interestingly, when the inhibitor was added after multicellular spheroid formation (S spheroid+IWP2), the mRNA expressions of *PCNA*, *CTNNB1*, *ITGAV*, *ITGA2*, *ITGB3*, *MMP14*, *WLS*, and *TGFB3* genes were all significantly changed to the similar levels as dissociated single hASCs (Single), demonstrating the unique and important role of Wnt signaling pathway in the regulation of hASC spheroid functions (Figure 7d). When S spheroid was treated with ICG001, an inhibitor of β‐catenin/TCF mediated transcription, the supernatant condition medium only resulted in the formation of smaller endothelial tubes on the Matrigel (Figure S17a,b, Supporting Information). Moreover, ICG001 treatment also significantly lowered the mRNA expression of hepatic genes (*ALB*, *AFP*, *CEBPA*, and *CYP3A4*) and typical albumin level (Figure S17c,d, Supporting Information). Hence, the canonical Wnt/β‐catenin signaling pathway contributed to the functional enhancements of hASC spheroids (Figure S17e, Supporting Information).

### 2.4 Co‐Culture of hASCs with HUVECs

#### 2.4.1 Randomly Distributed HUVECs

Due to the importance of vascularization for large tissues, we first co‐cultured hASCs with randomly distributed HUVECs in the PLdECM hydrogels and investigated the influence of HUVECs/hASCs seeding density ratio on hepatic differentiation (Figure S18a, Supporting Information). After hepatic induction, it was seen that co‐culture of randomly distributed HUVECs with hASCs significantly enhanced the mRNA expression of hepatocyte‐related genes (*ALB*, *CYP3A4*, *AFP*, *HNF4A*, *CK18*, *CEBPA*, *TBX3*, and *HGF*) (Figure S18b, Supporting Information). Meanwhile, the impact highly relied on the HUVECs/hASCs seeding density ratio, of which 2:1 showed the superior gene expression at mRNA (*ALB*, *CYP3A4*, *AFP*, *HNF4A*, *CK18*, *CEBPA*, *TBX3*, and *HGF*) and protein (HNF‐4α) levels compared to the other counterparts (Figure S18b,c, Supporting Information). The multicellular architecture of hASC spheroids was retained during hepatic differentiation in the co‐culture system (Figure S18c, Supporting Information). To distinguish the contribution of co‐cultured HUVECs from that of multicellular hASC spheroids to hepatic differentiation, hASCs were either cultured as cell spheroids (hASC spheroids) or dissociated single cells alone (Single hASCs) or with randomly distributed HUVECs at a HVECs/hASCs seeding density ratio of 2:1 (HUVECs/Single hASCs 2:1) in the PLdECM hydrogels (Figure S19a, Supporting Information). After hepatic induction in a series of cocktails, the mRNA expression of multiple hepatocyte‐related genes (*ALB*, *SOX9*, *CYP1A2*, *CYP3A4*, *AFP*, *CK18*, and *CEBPA*) was distinctly enhanced in hASC spheroids (Figure S19b, Supporting Information). Although co‐culture of dissociated single hASCs with HUVECs also increased the expression of partial genes (*ALB*, *SOX*, *CYP3A4*, *AFP*, and *CEBPA*), the improvement was not as effective as that induced by hASC spheroids for some genes (*ALB*, *CYP1A2*, *AFP*, *CK18*, *CEBPA*, and *HNF4A*) (Figure S19b, Supporting Information). The differential gene expression was also evidenced in the immunofluorescent staining of HNF‐4α (Figure S19c, Supporting Information). Even cultured and induced differentiation in the PLdECM hydrogels for weeks, hASC spheroids still kept multicellular architectures (Figure S19d, Supporting Information).

#### 2.4.2 Hexagonally Patterned HUVECs

To highlight the benefits of liver lobule‐like structure, HUVECs were either randomly distributed or hexagonally patterned by single extrusion‐based 3D bioprinting before homogeneously mixed with the dissociated single hASCs (Figure S20a, Supporting Information). Interestingly, hASCs co‐cultured with 3D‐printed HUVECs showed significantly higher levels of *ALB*, *HGF*, *CEBPA*, *TBX3*, *CYP3A4*, *SOX9*, and *CK18* gene expression than that with randomly distributed HUVECs (Figure S20b, Supporting Information). Moreover, immunofluorescent staining of albumin further confirmed the premium role of such bioinspired vascularized hexagons in the hepatic differentiation of hASCs (Figure S20c, Supporting Information). The 3D cell patterning resulted in a higher density of local HUVECs and herein increased their interaction with hASCs during cell co‐culture and differentiation (Figure S20d, Supporting Information).

#### 2.4.3 Hexagonally Patterned Hollow Channels

In addition, we also constructed hollow channels hexagonally patterned in the PLdECM hydrogels and investigated the influence of such special architecture on the hepatic induction of surrounding hASCs (Figure S21a, Supporting Information). Surprisingly, hepatic differentiation was more distinct for hASCs embedded in the PLdECM hydrogels with hexagonally patterned hollow channels as evidenced by relevant gene expression at mRNA (*AFP*, *CK18*, *CEBPA*, and *HGF*) and protein (albumin and HNF‐4α) levels (Figure S21b,c, Supporting Information).

#### 2.4.4 Hexagonally Patterned Hollow Vasculatures

To study the interactions between hASCs and HUVECs in a vascularized liver lobule‐like analog, the 3D‐printed HUVECs of hexagonally interconnected vasculatures were pre‐labeled with DiD dye, while the randomly distributed HUVECs were pre‐incubated with calcein‐AM before homogeneously mixing with small‐sized hASC spheroids in the parenchyma (Figure S22a,b, Supporting Information). After co‐cultured with hASC spheroids for 7 days, distinct blood capillaries were formed in the parenchyma of the vascularized liver lobule‐like analog with some invaded 3D‐printed HUVECs (Figure S22c, Supporting Information).

### 2.5 Ectopic Implantation of Vascularized Mini‐Livers for Rescue of CCl_4_‐Induced ALF

**Figure 8:**
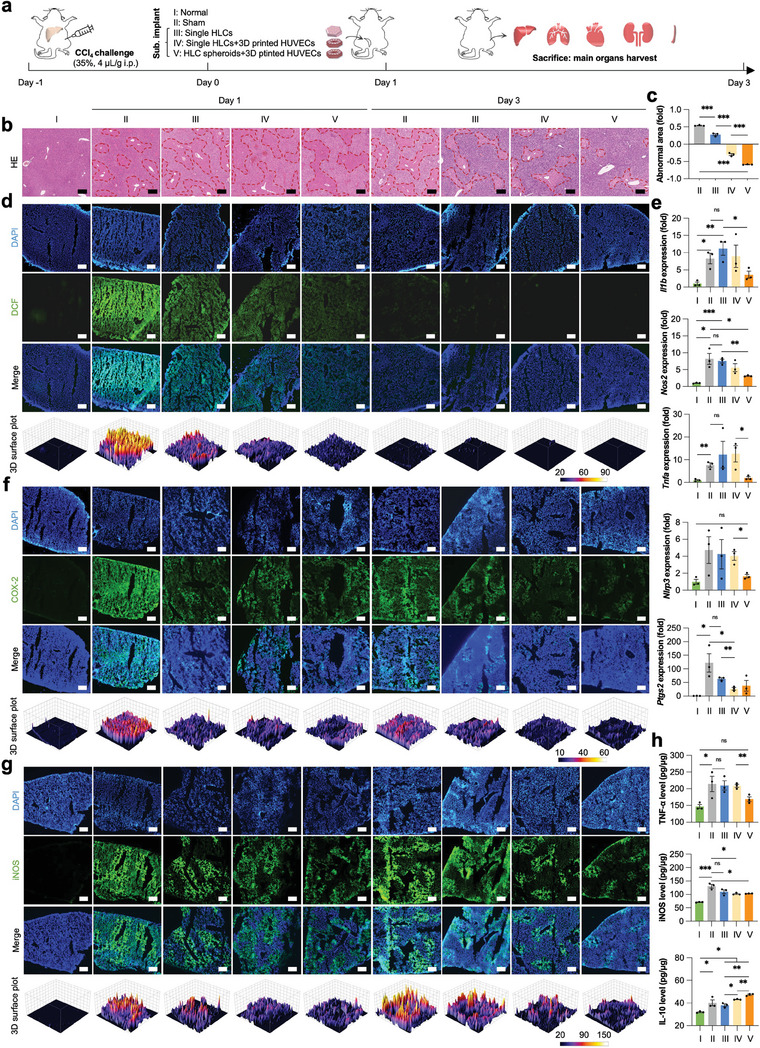
Treatment of CCl_4_‐induced ALF by vascularized hepatic lobule‐like analogs. a) A schematic image illustrating the procedure of animal experiments. b) H&E staining of mouse livers with different treatments on day 1 and 3. Blue: cell nuclei; Red: cytoplasm; Pink: collagen. Scale bar: 100 µm. Red dash lines show the abnormal areas. c) Quantification of the abnormal liver area changes according to the HE‐stained images by normalizing the total area on day 3 to that on day 1. d) The reactive species (ROS) levels in the mouse livers with different treatments. Green: ROS‐oxidized DCF; Blue: DAPI‐labeled cell nuclei. Scale bar: 200 µm. e) The mRNA expression of proinflammation‐related genes in the mouse livers with different treatments on day 1. All data are normalized to the value of “I: Normal” group. f‐g) Immunofluorescent staining of COX‐2 and iNOS in the mouse livers with different treatments. Green: Alexa Fluor 488‐labeled COX‐2 or iNOS; Blue: DAPI‐labeled cell nuclei. Scale bar: 200 µm. h) The protein levels of TNF‐α, iNOS, and IL‐10 in the mouse livers with different treatments characterized by ELISA kits on day 1. All data are normalized to the total protein. “I: Normal”: normal mice without challenging of carbon tetrachloride (CCl_4_); “II: Sham”: CCl_4_‐challenged mice without treatment; “III: Single HLCs”: CCl_4_‐challenged mice with subcutaneous implantation of single hASCs‐derived hepatocyte‐like cells (HLCs) in porcine liver‐derived decellularized extracellular matrix (PLdECM) hydrogel; “IV: Single HLCs+3D printed HUVECs”: CCl_4_‐challenged mice with subcutaneous implantation of single HLCs in PLdECM hydrogel and 3D printed HUVECs; “V: HLC spheroids+3D printed HUVECs”: CCl_4_‐challenged mice with subcutaneous implantation of HLC spheroids in PLdECM hydrogel and 3D printed HUVECs. The dosage of HLCs was kept consistent at 1 × 10^6^ cells/mouse. All data are presented as mean ± SEM. *n* = 3, 0.01 < *p < 0.05, 0.001 < **p < 0.01, and ***p < 0.001, not significant (ns) *p* > 0.05.

As small‐sized hASC spheroids equipped with 3D‐printed vasculatures had been evidenced with enhanced liver functions after induced hepatic differentiation in vitro, we believed the vascularized mini‐livers could be the alternatives to the limited liver donors for effective treatment of ALF. Due to the low cell survival in the harsh local microenvironment, we and others have previously shown that ectopically implanted liver mimics could increase the survival ratio of ALF‐challenged animal models, ameliorate oxidative stress and inflammation, and herein advance liver regeneration.^[^ ^10^, ^12^ ^]^ Therefore, in this study we first subcutaneously implanted the biomimetic hepatic analogs near the inguinal fat pads with rich blood vessels to study their therapeutic efficacy in the treatment of CCl_4_‐induced ALF (**Figure** **8**a).

The areas of abnormal necrotic tissues significantly decreased 3 days after implantation of differentiated dissociated single hASCs (Single HLCs) in comparison with that on day 1 (Figure 8b,c). Although co‐culture of dissociated single hASCs with 3D‐printed hexagonal vasculatures and induction of hepatic differentiation in vitro (Single HLCs+3D printed HUVECs) further reduced the necrotic area of liver tissues after the 3‐day treatment, it was still less effective than the induced hASC spheroids equipped with 3D‐printed vasculatures (HLC spheroids+3D printed HUVECs) (Figure 8b,c). Accordingly, there were fewer apoptotic cells in the livers of ALF‐challenged mice after ectopic implantation of different formulae, especially for “HLC spheroids+3D printed HUVECs” group (Figure S23a, Supporting Information). In contrast, an increasing population of proliferative cells was shown in “HLC spheroids+3D printed HUVECs” groups, which was more prominent on day 3 (Figure S23b, Supporting Information). Of note, the reactive oxygen species (ROS) were decreasingly accumulated in “HLC spheroids+3D printed HUVECs” groups as early as 1‐day post‐transplantation (Figure 8d).

1‐day post‐transplantation, the mRNA expression of pro‐inflammatory genes (*Il1b*, *Nos2*, *Tnfa*, and *Nlrp3*) was significantly decreased in the livers of “HLC spheroids+3D printed HUVECs” group as compared to all other groups, while only *Ptgs2* gene was distinctly downregulated in the “Single HLCs+3D printed HUVECs” group (Figure 8e). Immunofluorescent staining of inflammation‐related factors (COX‐2, iNOS, and IL‐10) and inflammatory cell marker (Ly‐6G) also demonstrated the consistently superior alleviation of inflammation storm in “HLC spheroids+3D printed HUVECs” group compared to other treatment groups up to 3 days after implantation (Figure 8f,g; Figure S24, Supporting Information). Similarly, western blots and ELISA quantification further confirmed the effective reduction of pro‐inflammatory factors (TNF‐α and iNOS) and the increase of anti‐inflammatory factor (IL‐10) (Figure 8h; Figure S25, Supporting Information). 3‐day post‐transplantation, there was no visible abnormal nodule in the other main organs, including the heart, lungs, kidneys, and spleen (Figure S26, Supporting Information).

### 2.6 Ectopic Implantation of Vascularized Mini‐Livers for Rescue of APAP‐Induced ALF

**Figure 9:**
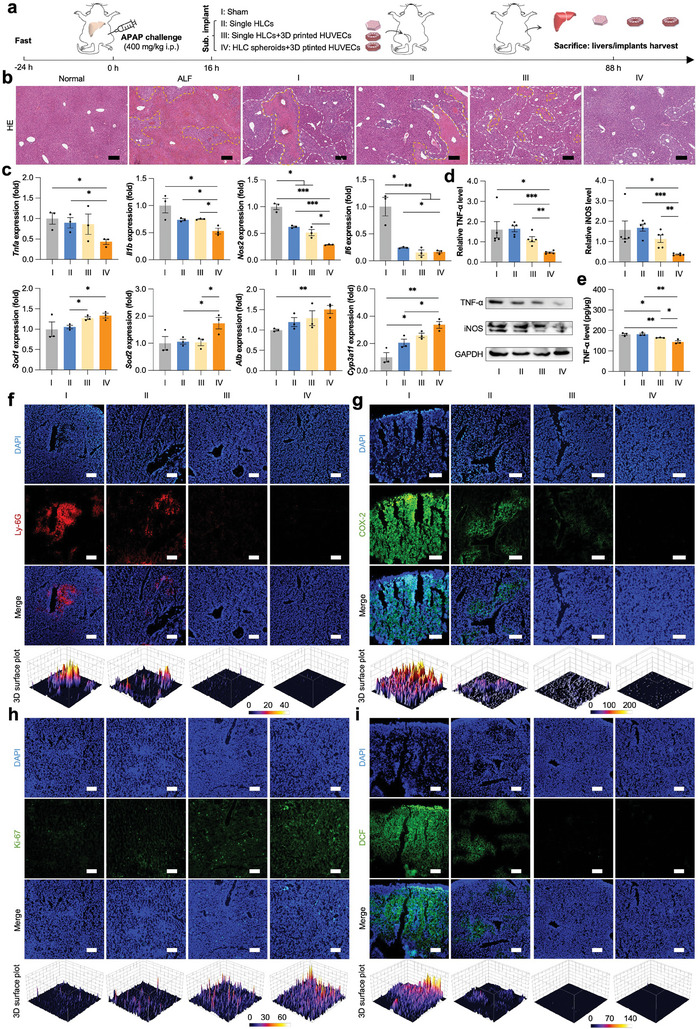
Treatment of APAP‐induced ALF by vascularized hepatic lobule‐like analogs. a) A schematic image illustrating the procedure of animal experiments. b) H&E staining of mouse livers with different treatments at 16 or 88 h. Blue: cell nuclei; Red: cytoplasm; Pink: collagen. Yellow dashed lines highlight the hyperemic area. White dashed lines indicate the infiltration of inflammatory cells. c) The mRNA expression of proinflammation‐related genes (*Tnfa*, *Il1b*, *Nos2*, and *Il6*), antioxidative genes (*Sod1* and *Sod2*), and hepatic function‐related genes (*Alb* and *Cyp3a11*) in the mouse livers with different treatments. All data are normalized to the value of “Sham” group and presented as mean ± SEM, *n* = 3. d) The western blots of TNF‐α and iNOS in the mouse livers with different treatments, and their quantification by Image J. GAPDH was used as the reference. All data are normalized to the value of GAPDH and presented as mean ± SEM, *n* = 5. e) The protein level of TNF‐α in the mouse livers with different treatments characterized by an ELISA kit. All data are normalized to the total protein and presented as mean ± SEM, *n* = 3. f–h) Immunofluorescent staining of Ly‐6G, COX‐2, and Ki‐67 in the mouse livers with different treatments. Green: Alexa Fluor 488‐labeled COX‐2 or Ki‐67; Red: PE‐labeled Ly‐6G; Blue: DAPI‐labeled cell nuclei. i) The reactive species (ROS) levels in the mouse livers with different treatments. Green: ROS‐oxidized DCF; Blue: DAPI‐labeled cell nuclei. “Normal”: normal mice without challenging of acetaminophen (APAP); “ALF”: APAP‐challenged mice at 16 h without treatment; “Sham”: APAP‐challenged mice without treatment at 88 h; “Single HLCs”: APAP‐challenged mice with subcutaneous implantation of single hASCs‐derived hepatocyte‐like cells (HLCs) in porcine liver‐derived decellularized extracellular matrix (PLdECM) hydrogel at 88 h; “Single HLCs+3D printed HUVECs”: APAP‐challenged mice with subcutaneous implantation of single HLCs in PLdECM hydrogel and 3D printed HUVECs at 88 h; “HLC spheroids+3D printed HUVECs”: APAP‐challenged mice with subcutaneous implantation of HLC spheroids in PLdECM hydrogel and 3D printed HUVECs at 88 h. The dosage of HLCs was kept consistent at 1 × 10^6^ cells/mouse. 0.01 < *p < 0.05, 0.001 < **p < 0.01, and ***p < 0.001, not significant (ns) *p* > 0.05. Scale bar: 200 µm.

In addition to the chemical‐induced ALF, we also subcutaneously implanted the vascularized biomimetic constructs near the inguinal fat pads of APAP‐challenged mice to investigate their therapeutic efficacy in the treatment of drug‐induced ALF (**Figure** **9**a). Compared to the “Sham” group, ectopic cell transplantation advanced the recovery of body weight in APAP‐challenged mice (Figure S27a, Supporting Information). 3‐day post‐transplantation, the representative liver in “HLC spheroids+3D printed HUVECs” group showed similar morphology to the normal counterpart, but in the absence of hyperemia, hardened texture, and visible necrotic speckles (Figure S27b, Supporting Information). Accordingly, HE staining of liver tissues also demonstrated the superior amelioration of hyperaemia and inflammatory cell infiltration in “HLC spheroids+3D printed HUVECs” group (Figure 9b). Co‐culture of dissociated single hASCs with 3D‐printed hexagonal vasculatures and induced hepatic differentiation in vitro highly enhanced the antioxidation and P450 enzyme activities after ectopic implantation into APAP‐challenged mice as evidenced by mRNA expression of *Sod1* and *Cyp3a11* genes (Figure 9c). Although *Nos2* and *Il6* genes were significantly downregulated in all treatment groups, “HLC spheroids+3D printed HUVECs” group showed distinctly decreased pro‐inflammatory genes (*Tnfa*, *Il1b*, *Nos2*, and *Il6*) as well as enhanced antioxidative genes (*Sod1* and *Sod2*) and hepatocyte‐related genes (*Alb* and *Cyp3a11*) (Figure 9c). In addition, the immunomodulatory effects of the vascularized hepatic lobule‐like analogs (HLC spheroids+3D printed HUVECs) were also confirmed by measurement of TNF‐α and iNOS protein levels in the supernatants of lysed liver tissues via western blots and ELISA (Figure 9d,e). Both 3D‐printed hexagonal vasculatures and multicellular architecture contributed to the effective eradication of inflammation storm as evidenced by immunofluorescent staining of pro‐inflammatory factors (COX‐2 and iNOS) as well as inflammatory cell marker (Ly‐6G) (Figure 9f,g; Figure S27c, Supporting Information). The ROS in “Sham,” “Single HLCs,” “Single HLCs+3D printed HUVECs,” and “HLC spheroids+3D printed HUVECs” groups were decreasingly accumulated, while the proliferating cells showed increasing populations (Figure 9h,i).

### 2.7 Maintenance of Functional Enhancements in the Vascularized Mini‐Livers in vivo

Since the multicellular architecture of hASC spheroids and their co‐culture with hexagonally patterned interconnected vasculatures at a specific HUVECs/hASCs seeding density ratio have shown remarkable contributions to the hepatic differentiation and vascularization in vitro, we further characterized the biomimetic construct as a whole 3‐day post‐transplantation in vivo to recognize its therapeutic roles. It was shown that the mRNA expressions of hepatocyte‐related genes (*HNF4A*, *ALB*, *CEBPA*, *AFP*, *CYP3A4*, and *SOX9*) were still highly enhanced in the implanted HLCs equipped with 3D‐printed vasculatures, especially for those engineered into small‐sized multicellular architectures (Figure S28a, Supporting Information). Accordingly, the up‐regulated expression of HNF‐4α was confirmed by immunofluorescent staining (Figure S28b, Supporting Information). There were more proliferating cells in the subcutaneous implants equipped with 3D‐printed vasculatures compared to that in “Single HLCs” group (Figure S28c, Supporting Information). In addition, blood capillary‐like structures were more distinctly visible in the vascularized implants containing HLC spheroids by immunofluorescent staining of CD31, an endothelial cell marker (Figure S28d, Supporting Information).

## 3 Discussion

ALF is a devastating disease with a high fatality rate and requires timely medical interventions.^[^ ^2^, ^13^ ^]^ However, standard liver transplantation is often restricted by limited donors, and the conventional temporally supporting system, bioartificial liver (BAL), is bulky and complex.^[^ ^14^ ^]^ Although hepatocyte infusion has shown some promising results in the relief of some ALF symptoms, the harsh local environment could result in low cell survival and compromise the final therapeutic outcomes.^[^ ^15^ ^]^ We and others have previously shown that ectopically implanted hepatocytes could function as remote satellites for the amelioration of local oxidative stress and inflammation storm, leading to decreased tissue necrosis, improved cell proliferation, and increased animal survival rate.^[^ ^10^, ^12^ ^]^ Particularly, engineering hepatocytes into multicellular spheroids significantly improved their therapeutic efficacy.^[^ ^4^, ^10^, ^16^ ^]^ However, obtainment of sufficient normal autologous/allogenic hepatocytes was also challenging due to their difficult expansion and easy loss of hepatocyte‐related functions in vitro.^[^ ^17^ ^]^ Hence, stem cell‐derived HLCs become the emerging substitutes, among which MSC‐derived HLCs are of great interest due to their low immunogenicity, convenient access, no ethical issue, and tumorigenicity.^[^ ^14^ ^]^ We previously demonstrated that paracrine effects and cell differentiation were enhanced in the MSC spheroids compared to an equivalent number of dissociated single cells.^[^ ^18^ ^]^ However, whether cell spheroid size, within the nutrients and oxygen penetrating limit distance (≈200 µm), can affect the functional enhancements is unclear, not to mention the underlying mechanism.^[^ ^7^ ^]^ In this regard, we fabricated hASC spheroids with five different dimensions (approximate diameter of 25 µm (XS), 50 µm (S), 100 µm (M), 150 µm (L), 200 µm (XL)) on microwell arrays for comparison of their pro‐angiogenic effects and hepatic differentiation efficacy. It was shown that S spheroid significantly improved migration, proliferation, angiogenesis of endothelial cells (Figure 3; Figures S6,S10–S12, Supporting Information), highlighting the pro‐angiogenic effects of stem cell‐derived secretomes.^[^ ^19^ ^]^ Meanwhile, small‐sized hASC spheroids were also superior in hepatic differentiation (Figure 4; Figure S13, Supporting Information). Using transcriptomic sequencing analysis, we found the engagement of multiple signaling pathways in the functional enhancements of hASC spheroids compared to the dissociated single cells, among which Wnt signaling is particularly of importance since it is an evolutionary cell‐to‐cell coordination pathway that regulates a variety of biological processes, including cell polarity, proliferation, migration, and differentiation (Figure 5).^[^ ^20^ ^]^ A recent study revealed that hepatocyte functions could be improved via the activation of the Wnt/β‐catenin pathway.^[^ ^21^ ^]^ Similarly, we found that genes encoding Wnt molecules and β‐catenin were significantly upregulated in the S spheroid (Figure S16, Supporting Information). Meanwhile, the GSEA analysis revealed that Frizzled binding, an early procedure of Wnt signaling, was accordingly upregulated in the S spheroid (Figure 6c). When S spheroid was treated with IWP2 or ICG001, extensive gene changes were evidenced (Figure 7d; Figure S17, Supporting Information). Taken together, the size‐dependent functional differences among hASC spheroids might be attributed to the distinctly autocrine Wnt from S spheroid that activated Wnt signaling pathway and led to β‐catenin‐mediated transcription of genes involved in the pro‐angiogenesis and hepatic differentiation (Figure S17e, Supporting Information).^[^ ^22^ ^]^ The finding of size‐dependent functional enhancements of hASC spheroids might advance the clinical translation of hepatocyte therapy as fewer cells could achieve the similar or even better therapeutic efficacy.

To imitate the liver lobule structure, we fabricated a biomimetic construct by 3D coaxial bioprinting of HUVECs into hexagonal vasculatures, consisting of a central vein and interconnected sinusoids, and filling the intervals with small‐sized hASC spheroids‐loaded PLdECM hydrogel as the parenchyma.^[^ ^23^ ^]^ The use of thermosensitive PLdECM hydrogel for encapsulation of hASC spheroids was owing to their excellent maintenance of hepatocyte phenotypes and functions.^[^ ^4^, ^24^ ^]^ We found that the hepatic differentiation of hASC spheroids was more distinct when co‐cultured with HUVECs at a HUVECs/hASCs seeding density of 2:1 (Figure S18, Supporting Information), especially for hexagonally patterned HUVECs (Figure S20, Supporting Information). This might be because the premium pro‐angiogenic effects of hASC spheroids also increased gene expression of the essential components (HGF and FGF2) of inductive cocktails in the co‐cultured HUVECs, which in turn promoted hepatic differentiation of hASCs (Figures S6,S12, Supporting Information). A similar reciprocal interaction was also reported in other MSCs/HUVECs co‐culture systems.^[^ ^25^ ^]^ Studies have shown that biomimetic patterning of HUVECs promoted the maturation of stem cell‐derived HLCs, probably due to the recapitulation of in vivo cell–cell interactions.^[^ ^8^, ^26^ ^]^ In addition, the hexagonal hollow channels, to some extent, might facilitate the accessibility of inductive biomolecules to the embedded hASCs in the PLdECM hydrogels, thereby improving the cell differentiation (Figures S1c,S2,S21, Supporting Information).^[^ ^27^ ^]^ The interactions between hASCs and 3D‐printed HUVECs, during cell co‐culture and differentiation, highlighted the importance of vascularization and the necessity of biomimetic architectures when bioengineering a hepatic analog with proper liver functions.

We previously demonstrated that subcutaneously implanted hepatocyte spheroids could form neo‐vessels and anastomose with the host vasculatures for proper functioning.^[^ ^10^ ^]^ On one hand, such ectopic implants could accelerate the detoxification or metabolism of chemicals by cytochrome P450 enzymes. On the other hand, the daily liver functions could also be partially and temporally taken by the implanted hepatocytes to buy time for host liver regeneration. In the current study, we showed that the vascularized hepatic lobule‐like analogs could also effectively ameliorate oxidative stress and inflammation at the early stage of ALF and herein prevent ALF deterioration (Figures 8 and 9; Figures S23–S25,S27, Supporting Information). The therapeutic efficacy was successfully evidenced in CCl_4_‐induced and APAP‐induced ALF, indicating the clinical translation potential of such a strategy.

## 4 Conclusion

To treat the devastating ALF, we fabricated a vascularized mini‐liver by co‐culture of small‐sized hASC spheroids with 3D‐printed hexagonal vasculatures. Interestingly, the hASC spheroids showed size‐dependent enhancements of pro‐angiogenic effects and hepatic differentiation via the Wnt signaling pathway. Meanwhile, cell differentiation and vascularization were enhanced during cell co‐culture. Of importance, the ectopically implanted mini‐livers showed good therapeutic efficacy in the treatment of CCl_4_‐ and APAP‐ induced ALF, which was promising for future clinical translation.

## 5 Experimental Section

### Coaxial Printing of Hexagonal Liver Lobule‐Like Analogs

The hexagonal vasculatures were printed by a Bio‐architect SR 3D printer (Regenovo, China) using an external ink (1.5% sodium alginate (Aladdin, China), 5% gelatin (Sigma‐Aldrich, USA), and 1 mg mL^−1^ fibrinogen (Sigma‐Aldrich, USA) in 1× PBS (Biosharp, China)) and an internal ink (7.5% gelatin, 1.5% hyaluronic acid sodium salt (Aladdin, China), and 8 U mL^−1^ thrombin (Sigma‐Aldrich, USA) in 1× PBS). The external supporting ink and internal sacrificing ink were extruded through needles with a diameter of 0.8 and 0.26 mm, respectively, under specific air pressures (0.05 and 0.35 MPa for external and internal inks, respectively). Both inks were kept at 25 °C, while the needle tips were maintained at 20 °C and the temperature of the collecting plate was set at 4 °C. The hexagonal architectures, with a central hole (diameter of 1.5 mm), distributed number of 12, and a layer number of 4, were printed at a speed of 8 mm s^−1^. The 3D‐printed hexagonal vasculatures were subsequently immersed in a cold crosslinking solution containing 40 mm calcium chloride (CaCl_2_, Macklin, China), 0.9% sodium chloride (NaCl, Macklin, China), and 8 U mL^−1^ thrombin at 4 °C for 30 min. After crosslinking, the constructs were washed in warm 1 × PBS twice to remove the internal sacrificing ink and form hollow channels. The remaining hexagonal vasculature ink included 1.5% calcium‐crosslinked alginate and 1 mg mL^−1^ thrombin‐transformed fibrin. The porcine liver‐derived decellularized extracellular matrix hydrogels at a concentration of 10 mg mL^−1^ were prepared as the stuffing parenchyma ink according to the previous study.^[^ ^10^ ^]^

SPI and HVI were immersed in the same volume of 20 µg mL^−1^ collagenase II at 37 °C for up to 7 days. The remaining hydrogels were freeze‐dried overnight and weighted to calculate the degradation percentage according to the formula: Degradation (%) = (W_0_−W_i_)/W_0_ × 100%, where *W*_0_ represents the average initial dry weight of inks, and *W*_i_ is the dry weight of remaining inks on day 0, 1, 2, 3, and 7. The rhodamine B (Sigma‐Aldrich, USA) was incorporated in the 500 µL SPI or HVI at a concentration of 0.1 mg mL^−1^ and incubated in 1 mL 1× PBS at 37 °C. The cumulative releasing profiles were recorded for permeability study at different time points (0.5, 1, 2, 4, 8, 12, 24, 48, 72, 96, 120, and 144 h) by measuring the mean fluorescent intensities of the supernatant media using a Synergy H1 microplate reader (excitation/emission: 550 nm/578 nm) and normalization to the completely released value. To study the mechanical strength of each component, the crosslinked inks (HVI, SPI, and HVI/SPI 1:1) were respectively characterized by a HAAKE MARS modular advanced rheometer system (Thermo Scientific, USA) using the stress sweep mode at a constant frequency of 0.1 Hz or the frequency sweep mode at a constant stress of 0.1 Pa. The pro‐angiogenic potentials of SPI and HVI were assessed through tube formation and cell proliferation assays, using commercial Matrigel (BD, USA) as a positive control. Briefly, the overnight‐starved human umbilical vein endothelial cells (ATCC, USA) were pre‐stained in 0.1 µm calcein‐AM (Beyotime, China) at room temperature for 20 min and seeded on the 100 µL hydrogel‐coated 96‐well plates at a density of 4 × 10^4^ cells per well. The formed tubes were imaged using a Ti2‐U fluorescent microscope (Nikon, Japan) in 4 h and processed by image J software (NIH, USA) to calculate the relative total length. For cell proliferation assay, HUVECs were seeded in 100 µL different hydrogels at a density of 1 × 10^5^ cells mL^−1^ and measured by a CCK‐8 kit (Apexbio, USA) according to the manufacturer's instructions on day 1, day 3, day 5, and day 7. In addition, the 3D‐printed hollow channels and each crosslinked component were imaged by an EVO MA10 scanning electron microscope (SEM, Zeiss, Germany) at a voltage of 20 kV. The permeability of the 3D‐printed hollow hexagon was also assessed by monitoring the perfusion of 0.1 mg mL^−1^ rhodamine B into the channels for up to 30 min.

### Viability of 3D‐Printed Endothelial Cells

To study the biocompatibility of the inks and 3D printing process, HUVECs were embedded in the external ink at a density of 1 × 10^6^ cells mL^−1^ during 3D printing and cultured in a complete medium (DMEM/F12 1:1 basic medium (ThermoFisher, USA) supplemented with 10% fetal bovine serum (FBS, TBD, China) and 1× penicillin/streptomycin (Sigma‐Aldrich, USA)) at 37 °C with 5% of CO_2_ supply. The 3D‐printed constructs were incubated in a fresh basic medium containing 1 µm calcein‐AM and 1 µm propidium iodide (PI, MP Biomedicals, France) at room temperature for 20 min on day 1. After washing in 1 × PBS twice, the stained live and dead cells were imaged by a Ti2‐U fluorescent microscope.

### Generation of Different‐Sized Cell Spheroids

Briefly, different numbers of human adipose‐derived mesenchymal stromal/stem cells (SALIAI, China) (10k, 25k, 50k) were seeded onto the small‐sized magnetic polydimethylsiloxane (PDMS) microwell array, with a diameter of 5 mm containing ≈100 microwells and 4 mg mL^−1^ magnetic nanoparticle (MNPs) (dimension of each microwell: length × width × depth: 0.5 mm × 0.5 mm × 0.22 mm), and cultured in 100 µL complete medium containing 5% serum‐free additives (SALIAI, China) at 37 °C with 5% of CO_2_ supply as reported in the previous study.^[^ ^10^ ^]^ After overnight, different‐sized hASC spheroids (S spheroid, M spheroid, and L spheroid) were formed and harvested by centrifugation at 600 rcf. In addition, 10k dissociated hASCs were also seeded onto the plain PDMS microwell arrays with the same total area but different dimensions (extra‐small size (length × width × depth: 0.33 mm × 0.33 mm × 0.3 mm), small size, and extra‐large size (length × width × depth: 1.3 mm × 1.3 mm × 0.9 mm)) to produce cell spheroids with a wider range (XS spheroid, S spheroid, and XL spheroid, respectively).

### Characterization of Different‐Sized hASC Spheroids

After cell spheroids were formed, the supernatant media were replaced by a staining solution (hASC basic medium supplemented with 1 µm calcein‐AM and 1 µm PI). The cell viability was assessed by a Ti2‐U fluorescent microscope. The average size of cell spheroids was measured by Image J software. To show the internal multicellular organization, cell spheroids were also embedded in OCT components (SAKURA, Japan) and sectioned onto slides with a thickness of 8 µm using a CM1950 Cryostat (Leica, Germany) for routine hematoxylin and eosin (HE) staining. The mRNA expression of cytoskeleton‐ (*ATCB*), cell adhesion‐ (*ITGA2*, *ITGB3*, *ITGAV*, and *CTNNB1*), extracellular matrix remodeling‐ (*MMP14* and *MMP3*), proliferation‐ (*PCNA*), paracrine effect‐related (*WLS*, *HIF1A*, *CXCL12*, *TGFB3*, *VEGFA*, *IGF* and *HGF*) genes in different‐sized hASC spheroids was characterized by RT‐qPCR with corresponding primer pairs (Table S1, Supporting Information). *GAPDH* was used as a reference gene. 10k dissociated hASCs harvested from the 2D tissue culture plate (TCP) were used as the control. Furthermore, systemically transcriptome analysis of hASCs either cultured as cell spheroids (S1 – S3 spheroid and L1 – L3 spheroid) or dissociated single cells (Single1 – Single3) was also performed following the service provider's procedures (Lc‐Bio Technology, China). The differentially expressed genes were determined when *p* < 0.05 in the significant comparisons using R package edgeR (https://bioconductor.org/packages/release/bioc/html/edgeR.html). The analysis of gene interactions was conducted using the STRING database (https://string‐db.org/), applying a minimum interaction score of 0.4. Subsequently, these interactions were visualized and reorganized using Cytoscape 3.9.1 software. The circle size indicates the interactive frequency and line thickness reflects the confidence of data support. The hub genes were determined according to their interaction frequency.

### Pro‐Angiogenic Potential of hASC Spheroids

After hASC spheroids were formed on the microwell arrays, the supernatant media were harvested and stored at −80 °C until further use. Approximately 1 × 10^5^ HUVECs were seeded onto each well of a 24‐well plate and starved in the low serum medium (DMEM/F12 1:1 basic medium supplemented with 0.2% FBS and 1× P/S). After overnight, a central scratch was performed on the bottom of each well using 1 mL pipette tip and gently washed with 1× PBS twice. The remaining cells were subsequently cultured in the hASC spheroid‐derived media for 24 or 36 h. Cell migration was characterized by the measurement of the blank areas at four different regions of the wells by Image J. The mRNA expression of receptor‐ (*CXCR4*, *CXCR7*, and *MET*), angiogenesis‐ (*ANGPT1*), proliferation‐ (*MKI67*), *migration‐* (*ID1*), and paracrine effect‐related (*WLS*, *FGF2*, *TGFB1*, and *HGF*) genes in the HUVECs were evaluated by RT‐qPCR using corresponding primer pairs (Table S1, Supporting Information) after culturing in the hASC spheroid‐derived supernatant media for 24 h. *GAPDH* was used as a reference gene for all the experiments performed in this study. Approximately 5k HUVECs were seeded onto each well of a 96‐well plate and starved in low serum media overnight, after which the original media were replaced with 100 µL hASC spheroid‐derived supernatants. The cell proliferation was measured at 24 and 72 h by CCK‐8 assay following the manufacturer's protocols. To reveal the paracrine effect of hASC spheroids on angiogenesis, 3 × 10^4^ HUVECs pre‐incubated in the low serum medium overnight were re‐suspended in 100 µL supernatant media of different‐sized hASC spheroids and seeded on the 100 µL Matrigel‐coated 96‐well plate (Corning, USA). The formed tubes were imaged at 4 and 8 h by a Ti2‐U microscope and the regions of interest were processed by Image J to measure angiogenesis‐related parameters. In addition, HUVECs were also treated by the supernatant media derived from the same number of dissociated single hASCs from 2D TCP and used as the control. The paracrine factors in the supernatant media of dissociated hASCs (Single) and hASC spheroids (S spheroid and L spheroid) were measured by a human angiogenesis array kit (R&D systems, USA) according to the manufacturer's instructions. Each sample was measured in duplicate and quantified by Image J. All values were normalized to the initial cell number.

### Hepatic Differentiation of Different‐Sized hASC Spheroids

2 × 10^5^ dissociated single hASCs or hASC spheroids with equivalent cell numbers were respectively embedded in 250 µL 10 mg mL^−1^ PLdECM hydrogels (pH 7.2). After gelation, the cells were fed with 500 µL induction media to initiate hepatic differentiation by inductive cocktails according to the previous study.^[^ ^28^ ^]^ Basically, there were three stages, including the first 2‐day incubation in the deprived medium (low glucose DMEM basic medium (ThermoFisher, USA) supplemented with 20 ng mL^−1^ epidermal growth factor (EGF, PeproTech, USA) and 10 ng mL^−1^ basic fibroblast growth factor (β‐FGF, PeproTech, USA) and the following one‐week induction in the medium containing with 20 ng mL^−1^ hepatocyte growth factor (HGF, PeproTech, USA), 10 ng mL^−1^ β‐FGF, and 0.61 g L^−1^ nicotinamide (NAM, Sigma‐Aldrich, USA), as well as the final 2‐week maturation in the medium supplemented with 20 ng mL^−1^ oncostatin M (OSM, PeproTech, USA), 1 µm dexamethasone (Dex, Solarbio, China), and 1× ITS additives (Cyagen, USA). The mRNA of induced hASCs was extracted to measure the expression of hepatocyte‐related genes (*ALB*, *CYP3A4*, *AFP*, *CK18*, *CEBPA*, and *SOX9*) with corresponding primer pairs (Table S1, Supporting Information) by RT‐qPCR.^[^ ^16^ ^]^ *GAPDH* was used as a reference gene. The cell‐gel hybrid constructs were embedded in OCT components and sectioned onto slides with a thickness of 8 µm for routine HE staining. Moreover, the relevant proteins, including E‐cadherin, albumin, and hepatocyte nuclear factor 4 alpha (HNF‐4α), were detected by immunofluorescent staining with the rabbit‐anti‐human primary antibodies (1:200 dilution for E‐cadherin, 1:100 dilution for albumin, 1:250 dilution for HNF‐4α, all from ThermoFisher, USA) and the Alexa Fluor 488‐labeled goat‐anti‐rabbit secondary antibody (1:500 dilution, ThermoFisher, USA).

### The Underlying Mechanisms Involving Size‐Dependent Functional Enhancements of hASC Spheroids

hASCs were seeded either on the 2D TCP or small‐sized magnetic PDMS microwell arrays to generate dissociated single cells (Single) or multicellular spheroids (S spheroid and L spheroid) with ≈2 × 10^5^ cells per biological repeat. The Wnt production inhibitor (IWP2, Beyotime, China) was added before or after the formation of small‐sized hASC spheroids (IWP2 + S spheroid and S spheroid + IWP2, respectively) at a final concentration of 0.5 µm to study the involvement of the pathway during the formation of cell spheroids and thereafter functional enhancements. The mRNA of dissociated single cells and different‐sized cell spheroids was extracted in 24 h for comparison of representative gene expression (*ACTB*, *PCNA*, *CTNNB1*, *ITGAV*, *ITGA2*, *ITGB3*, *MMP14*, *WLS*, and *TGFB3*).

The implication of the Wnt/β‐catenin pathway in the pro‐angiogenic potential and hepatic differentiation of small‐sized hASC spheroids were further investigated using ICG001 (ICG, Beyotime, China), an inhibitor of β‐catenin/TCF mediated transcription. After small‐sized hASC spheroids with a diameter of ≈50 µm were formed overnight on the microwells, they were incubated with 5 µm ICG001 for another 24 h. The cells treated with DMSO were used as the positive control. Fresh media supplemented with dimethyl sulfoxide (DMSO, Sigma‐Aldrich, USA) or ICG001 were used as the negative controls. The supernatant media were harvested for the tube formation assay on the Matrigel as described in the “Pro‐Angiogenic Potential of hASC Spheroids” section. After incubation for 8 h, the formed tubes were characterized by the mean mesh size. In addition, the hASC spheroids were resuspended in the PLdECM hydrogels and differentiated in the inductive cocktails as described in the “Hepatic Differentiation of Different‐Sized hASC Spheroids” section with an extra supplementary of DMSO or ICG001. After hepatic induction, the mRNA expression of hepatic genes (*ALB*, *AFP*, *CEBPA*, and *CYP3A4*) was characterized by RT‐qPCR, and the albumin level was evidenced by immunofluorescent staining.

### Hepatic Differentiation of hASC Spheroids Co‐Cultured with HUVECs at Different Cell Density Ratios

To study the interaction between HUVECs and hASC spheroids, HUVECs were co‐cultured with S spheroid in 200 µL PLdECM hydrogels at various density ratios (HUVECs : hASCs 0:1, 1:1, 2:1, 4:1). The number of seeded hASCs was kept consistent at 1.5 × 10^5^ cells. After gelation at 37 °C for 1 h, 400 µL induction medium was supplied to initiate hepatic differentiation. The mRNA expression of hepatocyte‐related genes (*ALB*, *CYP3A4*, *AFP*, *HNF4A*, *CK18*, *CEBPA*, *TBX3*, and *HGF*) was measured by RT‐qPCR using the corresponding primer pairs (Table S1, Supporting Information) after hepatic induction. The multicellular morphology and the HNF‐4α expression level of differentiated hASC spheroids were characterized by routine HE staining and immunofluorescent staining after frozen section, respectively.

To further compare the impacts of endothelial cell coculture and multicellular structure on the hepatic differentiation efficacy of hASCs, 1.5 × 10^5^ dissociated single hASCs were embedded in 200 µL PLdECM hydrogels with/without the coculture of HUVECs at a density ratio of 2:1 (HUVECs:hASCs). In addition, the equivalent number of hASCs were also engineered as S spheroid and cultured alone in the PLdECM hydrogels. The hepatic differentiation efficacy was characterized through the measurement of hepatocyte‐related gene expression at mRNA (*ALB*, *CYP3A4*, *AFP*, *HNF4A*, *CK18*, *CEBPA*, *CYP1A2*, and *SOX9*) and protein (HNF‐4α) levels. Moreover, hASCs were also stained by HE to show cell morphology after hepatic induction.

### Hepatic Differentiation of hASCs Co‐Cultured with Hexagonally Patterned HUVECs

To investigate the benefits of biomimetic liver lobule‐like hexagonal structure for the hepatic differentiation of hASCs, HUVECs were loaded into a 3D printing ink, consisting of 1.5% hyaluronic acid sodium salt, 1.5% sodium alginate, and 2.5% gelatin, and extruded onto a cold collecting plate through a 23G needle with an air pressure of 0.3 MPa and a moving speed of 8 mm s^−1^. The needle tip, syringe, and the collecting plate were kept at 20, 25, and 4 °C, respectively. The 3D‐printed liver lobule‐like hexagonal constructs composed of four layers of 3 mm length of sides and a 7.5 mm diameter of the central hole were crosslinked in the cold 40 mM CaCl_2_/0.9% NaCl solution for 30 min. After being washed in warm 1× PBS twice, the intervals of 3D‐printed hexagonal skeletons were filled with 200 µL 2 × 10^5^ hASCs‐embedded PLdECM hydrogels. The co‐culture blocks were incubated at 37 °C for 1 h to initiate gelation before the addition of 400 µL hepatic differentiation medium on the top. The randomly distributed HUVECs co‐cultured with hASCs at the same cell seeding density ratio (HUVECs:hASCs 2:1) in the hybrid gel (10 mg mL^−1^ PLdECM/1.5% alginate 1:1) were used as the control. Gelation was initiated at 37 °C for 1 h before calcium crosslinking. After additional two rounds of wash in 1× PBS, hASCs were induced hepatic differentiation. The mRNA expression levels of hepatocyte‐related genes (*ALB*, *CYP3A4*, *AFP*, *TBX3*, *CK18*, *CEBPA*, *HGF*, and *SOX9*) were characterized by RT‐qPCR and the protein levels of albumin and CD31 were evidenced by immunofluorescent staining.

### Hepatic Differentiation of hASCs in PLdECM Hydrogels with 3D‐Printed Hollow Channels

The liver lobule‐like hexagonal constructs with interconnected hollow channels were fabricated as described in the “3D Coaxial Printing of Hexagonal Liver Lobule Analogs” section but in the absence of HUVECs. 2 × 10^5^ dissociated single hASCs were embedded in 200 µL PLdECM hydrogels and cast into the intervals of 3D‐printed hexagonal hollow channels (hexagonal channels) for subsequent hepatic differentiation. The equivalent number of hASCs differentiated into hepatocyte‐like cells in the PLdECM hydrogels without 3D‐printed hollow channels (no‐hexagonal channels) were used as the control. The expression of hepatocyte‐related genes at mRNA (*AFP*, *CK18*, *CEBPA*, and *HGF*) and protein (albumin and HNF‐4α) levels was detected by RT‐qPCR and immunofluorescent staining, respectively.

### Vascularization of the 3D‐Printed Liver Lobule‐Like Analogs

After being starved in the low FBS medium overnight, 4 × 10^5^ HUVECs cultured on the 2D TCP were first stained by 1 µm calcein‐AM and embedded in 200 µL PLdECM hydrogels either with or without co‐culture of S spheroid at a HUVECs/hASCs density ratio of 2:1. Furthermore, another batch of HUVECs prelabeled with 5 µm DiD (Beyotime, China) were 3D printed into hexagonal vasculatures with interconnected hollow channels, of which the intervals were filled with the small‐sized hASC spheroids and calcein‐AM‐labeled HUVECs mixtures. The cells were cultured in 400 µL hybrid medium (hASCs complete medium : HUVECs low FBS medium 1:1) for up to 7 days with medium change twice per week. The morphology of HUVECs was recorded daily by a Ti2‐U fluorescent microscope.

### Animal Experiments

Balb/c (60) female mice (8‐week‐old, 18–21 g) were purchased from SJA Laboratory Animal (Guangzhou, China) and raised in a SPF environment with 12 h intervals of light and free access to food and water. All procedures in the animal experiments were reviewed and approved by the Institutional Animal Care and Use Committee of Sun Yat‐Sen University (Approval No. SYSU‐IACUC‐2023‐000621). Half of the mice were used for CCl_4_‐induced acute liver failure experiment, while the remaining mice were challenged by APAP. The mice were randomly distributed into five groups (Group A‐E, *n* = 6 for each group). Group A was the healthy control (Normal). The mice in Group B‐E were induced ALF by intraperitoneal injection (i.p.) of 35% CCl_4_ (Macklin, China) in olive oil (Macklin, China) or 10 mg mL^−1^ APAP (Sangon Biotech, China) in 1× PBS at a dosage of 4 or 40 µL per gram of mouse weight, respectively. The mice were fasted for 24 h before administration of APAP. Group B was the sham control with ALF challenging and fake surgery but in the absence of implants (Sham). The ALF‐challenged mice in Group C‐E were subcutaneously implanted with different formulae near the right inguinal fat pads where there are rich in blood vessels after i.p. anesthetization by 0.6% pentobarbital sodium (Sigma‐Aldrich, USA) in 1× PBS at a dosage of 10 µL per mouse weight after 24 h for CCl_4_‐challenged mice or 16 h for APAP‐challenged mice (Group C: 1 × 10^6^ dissociated single HLCs in 200 µL PLdECM hydrogels (Single HLCs); Group D: 3D‐printed vascularized hepatic lobule‐like analog consisting of HUVECs and 1 × 10^6^ dissociated single HLCs at a HUVECs/HLCs density ratio of 2:1 (Single HLCs+3D printed HUVECs); Group E: 3D‐printed vascularized hepatic lobule‐like analog consisting of HUVECs and HLC spheroids with equivalent cell number to Group D and a HUVECs/HLCs density ratio of 2:1 (HLC spheroids+3D printed HUVECs)). The 1 cm incision was closed by absorbable PGA 4‐0 sutures (Jinbei, China), and the mice were further fed for up to 3 days with daily weight record.

The CCl_4_‐challenged mice were sacrificed 1‐and 3‐day post‐implantation for main organ harvest and evaluation (liver, heart, lungs, spleen, and kidneys), while the mice challenged by APAP were anesthetized to collect livers and implants 3 days after ectopic cell therapy. The harvested main organs were processed for HE staining to show the safety of the mini‐livers. The necrosis of liver tissues was characterized by the necrotic area change from 1‐day post‐implantation to day 3 based on the HE‐stained images. Furthermore, the livers were also prepared for frozen section into slides with a thickness of 8 µm and stained by a TUNEL kit (Beyotime, China) following the manufacturer's instructions. Meanwhile, cell proliferation of the sectioned livers was also detected by immunofluorescent staining of Ki‐67 using a rabbit‐anti‐mouse primary antibody at a dilution ratio of 1:500 (Proteintech, China) and an Alexa Fluor‐labeled goat‐anti‐rabbit secondary antibody (1:500 dilution ratio, ThermoFisher, USA). Partial livers were milled in TRIzon reagent (CWBIO, China) or RIPA lysis buffer (Beyotime, China) for subsequent detection of inflammation‐related gene expression at mRNA level (*Il1b*, *Nos2*, *Tnfa*, *Nlrp3*, and *Ptgs2*) using corresponding primer pairs (Table S2, Supporting Information) or protein level via ELISA (TNF‐α, iNOS, and IL‐10, all from ANRC, China), western blot (iNOS (rabbit‐anti‐mouse primary polyclonal antibody, 1:4000 dilution ratio) and TNF‐α (rabbit‐anti‐mouse primary polyclonal antibody, 1:1000 dilution ratio) detected by a HRP‐labeled goat‐anti‐rabbit secondary antibody (1:5000 dilution ratio), all from Proteintech, China) as well as immunofluorescent staining (COX‐2 (rabbit‐anti‐mouse primary polyclonal antibody, 1:500 dilution, Proteintech, China), iNOS (1:500 dilution ratio), IL‐10 (rabbit‐anti‐mouse primary polyclonal antibody, 1:500 dilution ratio, Proteintech, China), Ly‐6G (PE‐labeled rat‐anti‐mouse monoclonal antibody, 1:200 dilution ratio, Biolegend, USA)). The oxidative stress in the livers was characterized by mRNA expression of antioxidative genes (*Sod1* and *Sod2*) and hepatocyte‐related genes (*Alb* and *Cyp3a11*) using corresponding primer pairs (Table S2, Supporting Information) in addition to ROS staining (Beyotime, China) according to the manufacturer's protocols. The implants were harvested from the APAP‐challenged mice on day 3 for comparison of hepatocyte‐related gene expression at mRNA (*HNF4A*, *ALB*, *CEBPA*, *AFP*, *CYP3A4*, and *SOX9*) and protein (HNF‐4α) levels. In addition, cell proliferation and vascularization of the implants were characterized by immunofluorescent staining of Ki‐67 and CD31 (rabbit‐anti‐human primary polyclonal antibody, 1:500 dilution, Proteintech, China), respectively.

### Statistical Analysis

All experiments were performed in triplicate and data were presented as mean ± SEM. The significant differences among groups were evaluated by a two‐tailed student's *t*‐test using GraphPad Prism 9.5.1 software (USA). The symbols “*,” “**,” and “***” were used when 0.01 < *p* < 0.05, 0.001 < *p* < 0.01, and *p* < 0.001, respectively.

## Conflict of Interest

The authors declare no conflict of interest.

## Supporting information

Supporting Information

## Data Availability

The data that support the findings of this study are available from the corresponding author upon reasonable request.
