# Supplementary material for: 3D Printing of a Vascularized Mini‐Liver Based on the Size‐Dependent Functional Enhancements of Cell Spheroids for Rescue of Liver Failure
Source: Adv Sci (Weinh). 2024 Feb 21;11(17):2309899. doi: 10.1002/advs.202309899 (PMC11077657; doi:10.1002/advs.202309899)
Supplement: Supplementary file 1 — Supporting Information [file ADVS-11-2309899-s001.pdf]

ADVANCED SCIENCE
Open Access

Supporting Information

for *Adv. Sci.*, DOI 10.1002/advs.202309899

3D Printing of a Vascularized Mini-Liver Based on the Size-Dependent Functional Enhancements of Cell Spheroids for Rescue of Liver Failure

Jiabin Zhang, Xiaodie Chen, Yurong Chai, Chenya Zhuo, Yanteng Xu, Tiantian Xue, Dan Shao, Yu Tao and Mingqiang Li**

Supporting Information

3D Printing of a Vascularized Mini-Liver Based on the Size-Dependent Functional Enhancements of Cell Spheroids for Rescue of Liver Failure

Jiabin Zhang, Xiaodie Chen, Yurong Chai, Chenya Zhuo, Yanteng Xu, Tiantian Xue, Dan Shao, Yu Tao, and Mingqiang Li**

J. Zhang, C. Zhuo, Y. Xu, Y. Chai, X. Chen, T. Xue, Y. Tao, M. Li
Laboratory of Biomaterials and Translational Medicine
Center for Nanomedicine
The Third Affiliated Hospital
Sun Yat-sen University
Guangzhou 510630, China
E-mail: taoy28@mail.sysu.edu.cn; limq567@mail.sysu.edu.cn

D. Shao
Institute of Life Sciences
School of Medicine
South China University of Technology
Guangzhou 510006, China

J. Zhang, C. Zhuo, Y. Xu, Y. Tao, M. Li
Guangdong Provincial Key Laboratory of Liver Disease
Guangzhou 510630, China

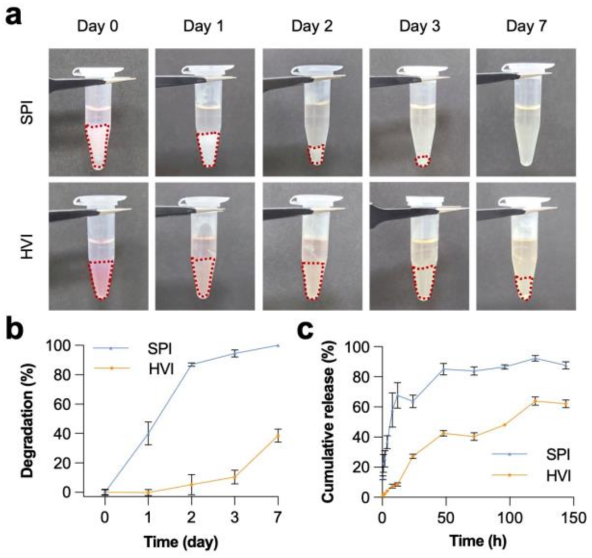

Figure S1. Degradation and permeability study of inks. (a) Optical images and (b) weight loss quantification of SPI and HVI in 20 μg mL^{-1} collagenase II at 37 ℃ at different time points (day 0, day 1, day 2, day 3, and day 7). The red dashed lines highlight the remaining hydrogels. All data are normalized to the initial weight and presented as mean ± SEM, *n* = 3. (c) The cumulative releasing profiles of rhodamine B from different inks. All data are normalized to the total value and presented as mean ± SEM, *n* = 5. SPI: stuffing parenchyma ink; HVI: hexagonal vasculature ink.

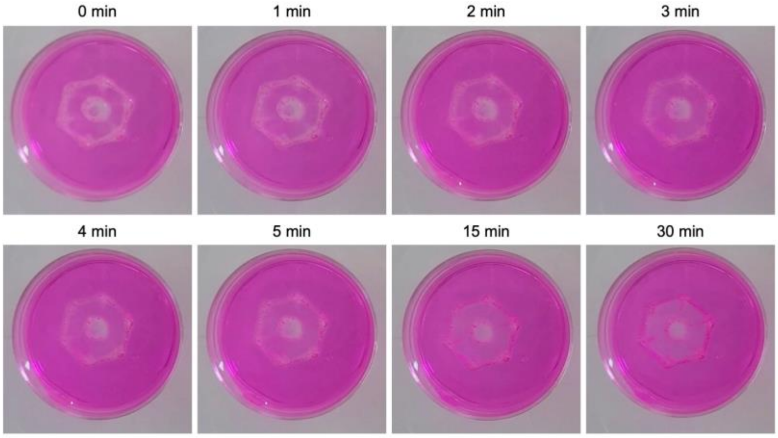

Figure S2. The representative images showing the perfusion of rhodamine B into the 3D-printed hollow hexagon at different time points.

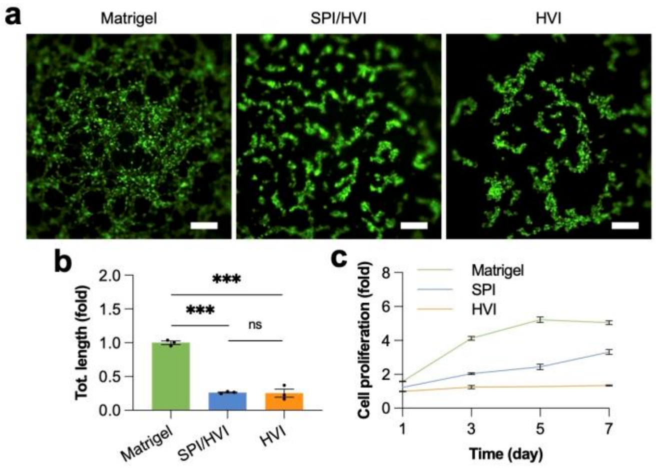

Figure S3. The pro-angiogenic potential of different hydrogels. (a-b) Fluorescent images of calcein AM-stained HUVECs on different substrates (Matrigel, SPI/HVI 1:1 mixture, or HVI) 4 h after cell seeding and total length of the formed meshes. All data are normalized to the value of the "Matrigel" group and presented as mean ± SEM, $n = 3$. ***$p < 0.001$, and not significant (ns) $p > 0.05$. Scale bar: 200 μm. (c) The relative cell proliferation of HUVECs cultured in different hydrogels. All data are normalized to the value of the "HVI" group on day 1 and presented as mean ± SEM, $n = 3$. SPI: stuffing parenchyma ink; HVI: hexagonal vasculature ink.

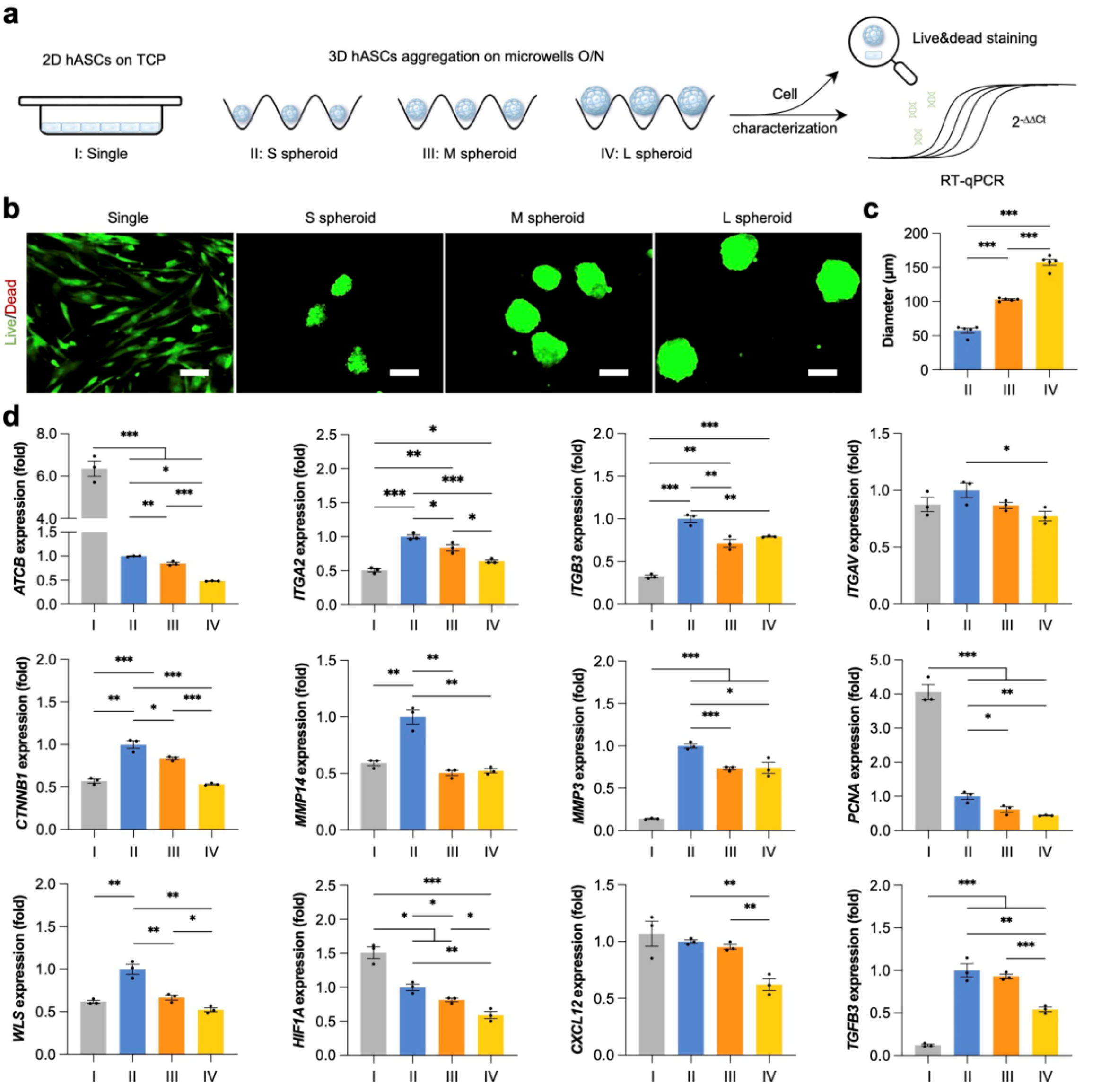

Figure S4. Characterization of hASC spheroids. (a) A schematic image illustrates the procedures for characterization of different-sized hASC spheroids. (b) Cell viability of hASCs cultured on different substrates at 24 h. Green: live cells; Red: dead cells. Scale bar: 100 μm. (c) Size quantification of hASC spheroids. Data are presented as mean ± SEM. $n = 5$. (d) The relative mRNA expression of representative genes in hASC spheroids with different sizes. Data are normalized to the value of “S spheroid” group and presented as mean ± SEM. $n = 3$. $0.01 < {}^{*}p < 0.05$, $0.001 < {}^{**}p < 0.01$, and ${}^{***}p < 0.001$, not significant (ns) $p > 0.05$.

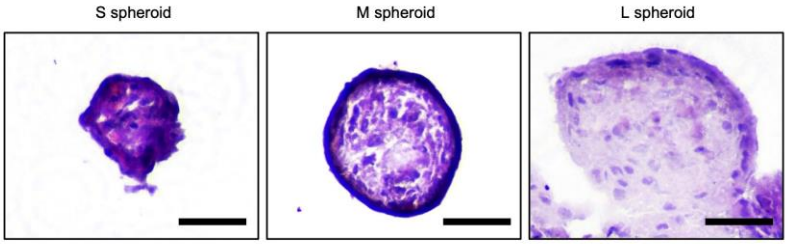

Figure S5. HE staining of hASC spheroids with different sizes. Blue: cell nuclei; Red: cytoplasm; Pink: collagen. Scale bar: 50 μm.

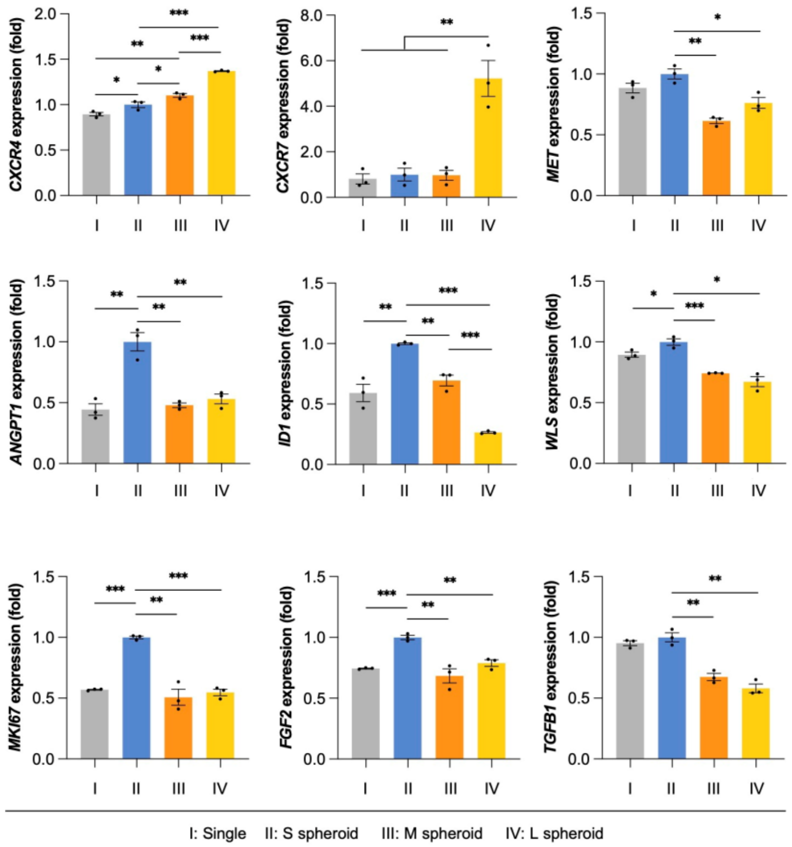

Figure S6. The relative mRNA expression of receptor-, angiogenesis-, proliferation-, migration-, and paracrine effect- related genes in HUVECs cultured with different hASC supernatant media for 24 h. All data are normalized to the value of "S spheroid" group and presented as mean ± SEM, $n = 3$, $0.01 < *p < 0.05$, $0.001 < **p < 0.01$, and $***p < 0.001$, not significant (ns) $p > 0.05$.

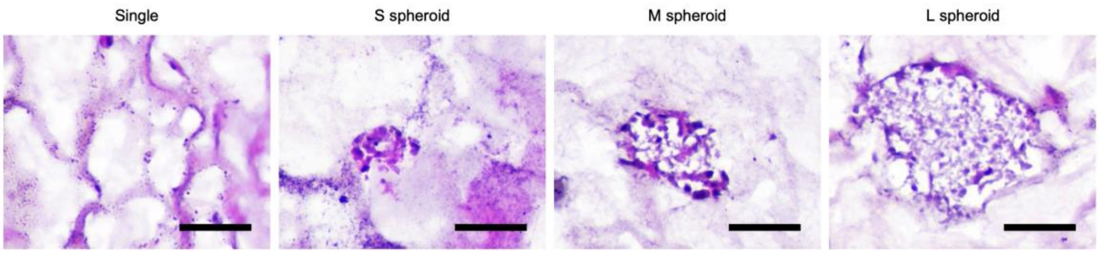

Figure S7. H&E staining of hepatic differentiation-induced hASCs either as single cells or cell spheroids with different sizes in PLdECM hydrogels. Blue: cell nuclei; Red: cytoplasm; Pink: collagen. Scale bar: 50 μm.

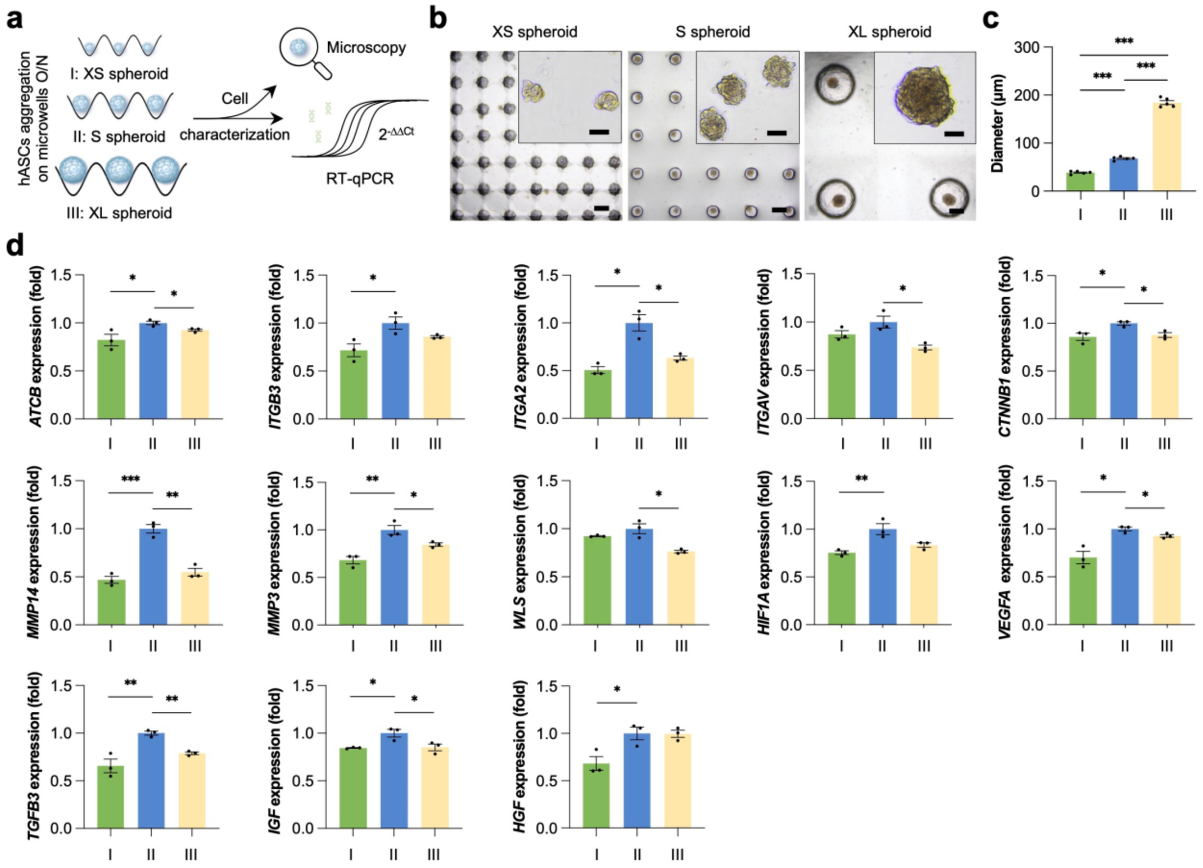

Figure S8. Characterization of hASC spheroids. (a) A schematic image illustrates the procedures for the characterization of different-sized hASC spheroids (XS spheroid, S spheroid, XL spheroid). (b) Optical microscopy images of formed hASC spheroids with distinct sizes using a seeding density of 10k cells per piece of microwell array in 24 h. Scale bar: 200 µm (inset scale bar: 50 µm). (c) Size quantification of hASC spheroids. Data are presented as mean ± SEM. $n = 5$. (d) The relative mRNA expression of representative genes in hASC spheroids with different sizes. Data are normalized to the value of "S spheroid" group and presented as mean ± SEM. $n = 3$. $0.01 < *p < 0.05$, $0.001 < **p < 0.01$, and $***p < 0.001$, not significant (ns) $p > 0.05$.

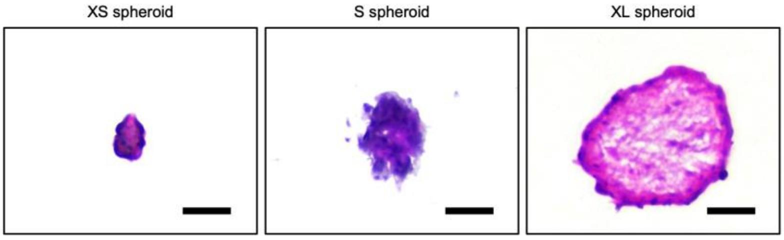

Figure S9. H&E staining of hASC spheroids with different sizes. Blue: cell nuclei; Red: cytoplasm; Pink: collagen. Scale bar: 50 μm.

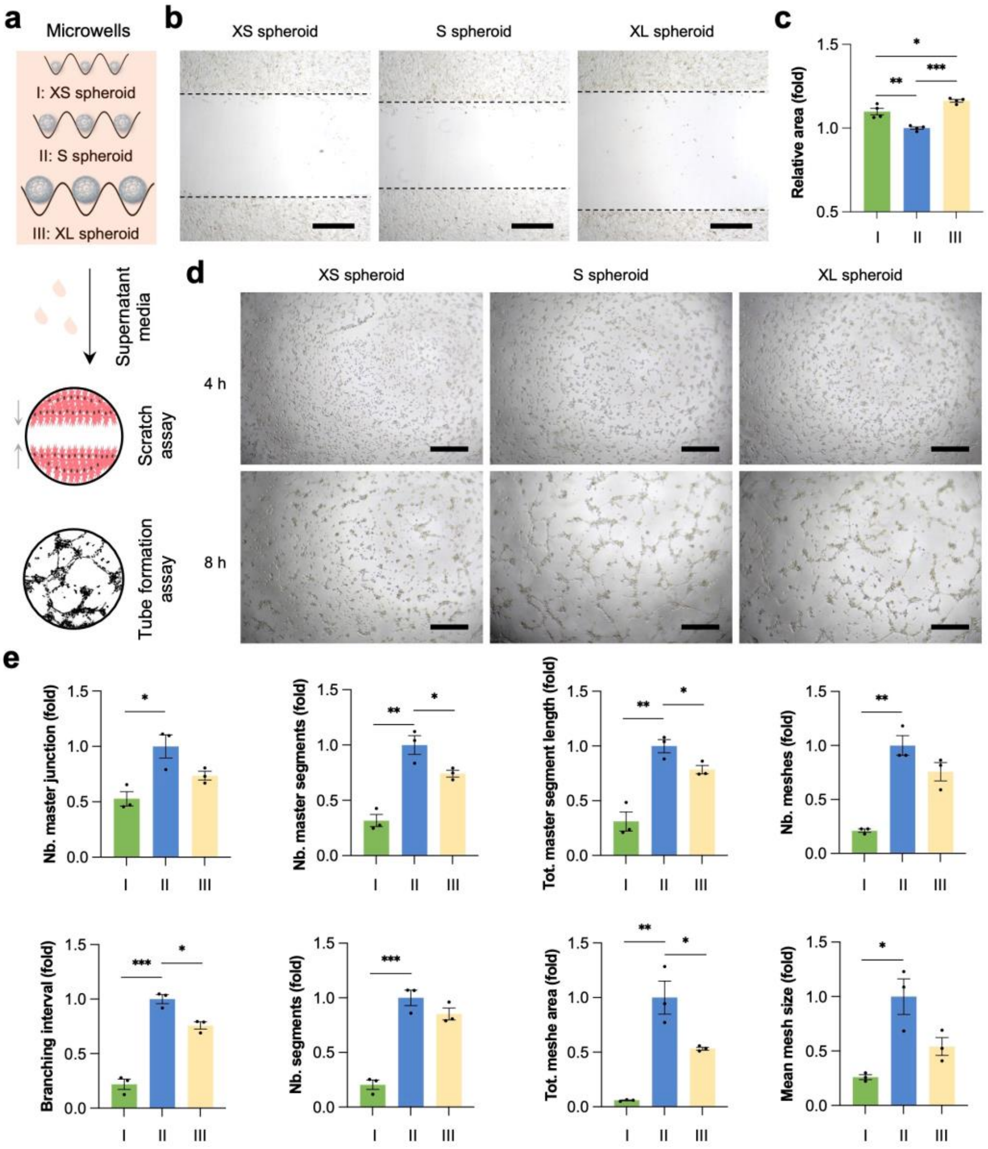

Figure S10. Pro-angiogenic effects of different-sized hASC spheroids. (a) A schematic image illustrates the procedures for studying the proangiogenic effects of hASC spheroids-derived secretome. (b) Migration of HUVECs cultured with different-sized hASC spheroids-derived supernatant media for 24 h. (c) The quantification of the remaining area. All data are normalized to the value of "S spheroid" group and presented as mean ± SEM, $n = 4$. (d) Tube formation of HUVECs cultured with different hASC supernatant media at 4 h and 8 h. (e) The quantification of the formed tubes at 8 h. All data are normalized to the value of "S spheroid" group and presented as mean ± SEM, $n = 3$. $0.01 < *p < 0.05$, $0.001 < **p < 0.01$, and $***p < 0.001$, not significant (ns) $p > 0.05$. Scale bar: 500 μm.

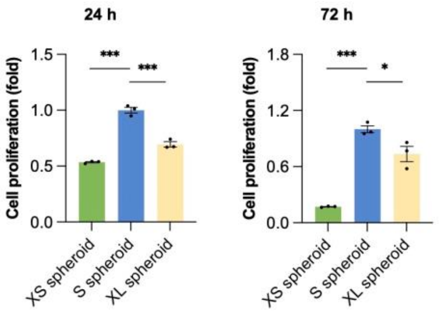

Figure S11. HUVEC proliferation at 24 h and 72 h. All data are normalized to the value of "S spheroid" group and presented as mean ± SEM, $n = 3$, 0.01 < *p < 0.05, 0.001 < **p < 0.01, and ***p < 0.001, not significant (ns) $p > 0.05$.

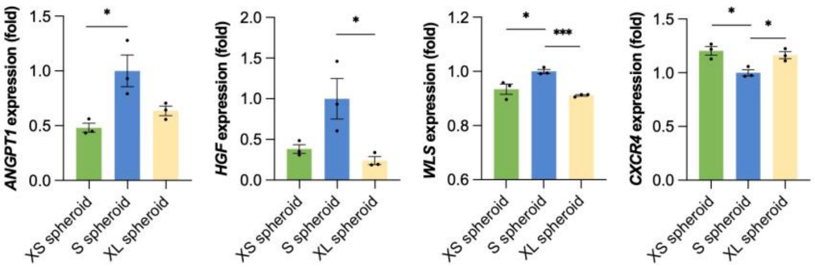

Figure S12. The relative mRNA expression of angiogenesis-, migration-, and paracrine effect-related genes in HUVECs cultured with different hASC supernatant media for 24 h. All data are normalized to the value of "S spheroid" group and presented as mean ± SEM, $n = 3$, 0.01 < *p < 0.05, 0.001 < **p < 0.01, and ***p < 0.001, not significant (ns) p > 0.05.

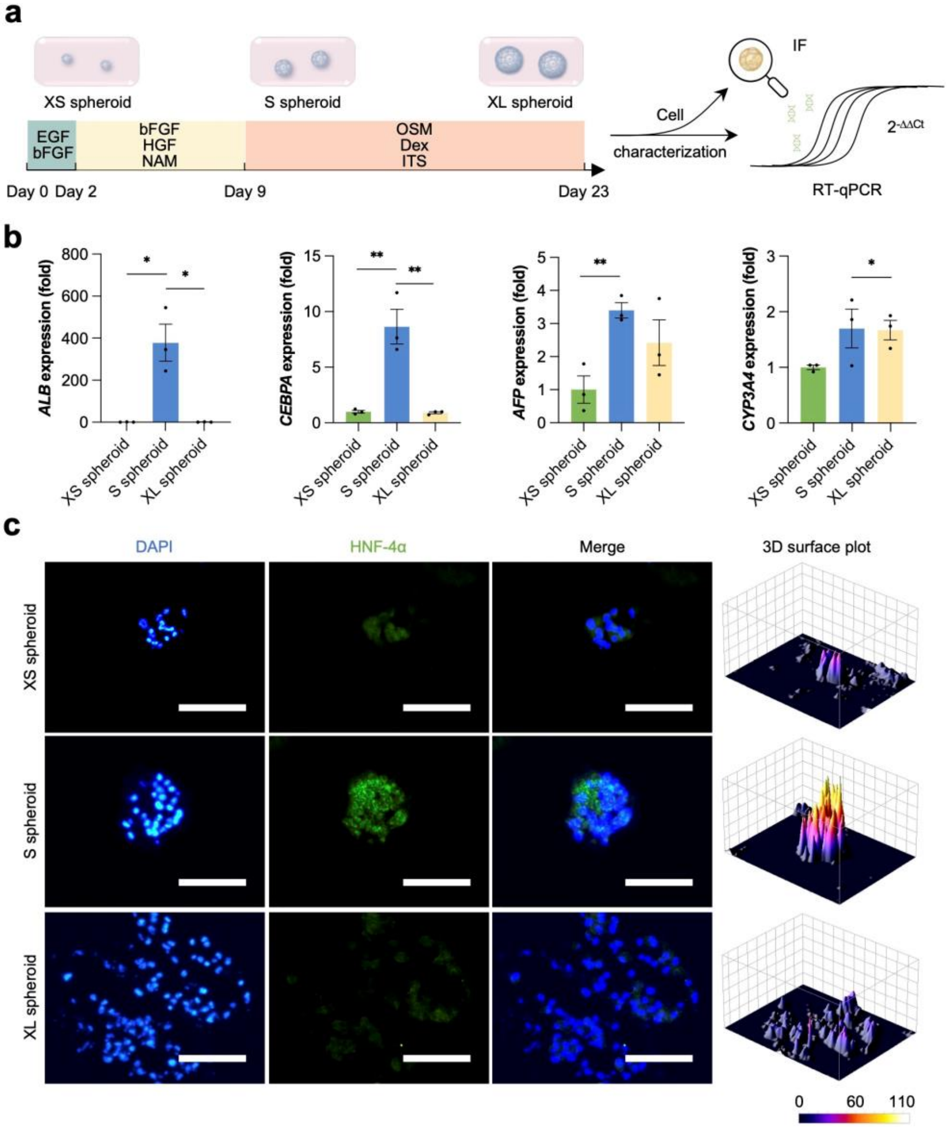

Figure S13. Hepatic differentiation of different-sized hASC spheroids. (a) A schematic illustrates the hepatic differentiation of different-sized hASC spheroids (XS spheroid, S spheroid, XL spheroid) in PLdECM hydrogels. (b) The relative mRNA expression of hepatic genes in hASCs cultured inside PLdECM hydrogels as cell spheroids with distinct sizes. All data are normalized to the value of "XS spheroid" group and presented as mean ± SEM, $n = 3$, $0.01 < *p < 0.05$, $0.001 < **p < 0.01$, and $***p < 0.001$, not significant (ns) $p > 0.05$. (c) Immunofluorescent staining of HNF-4α in hepatic differentiation-induced hASC spheroids with distinct sizes in PLdECM hydrogels. Green: Alexa Fluor 488-labelled HNF-4α; Blue: DAPI-labelled cell nuclei. Scale bar: 100 μm.

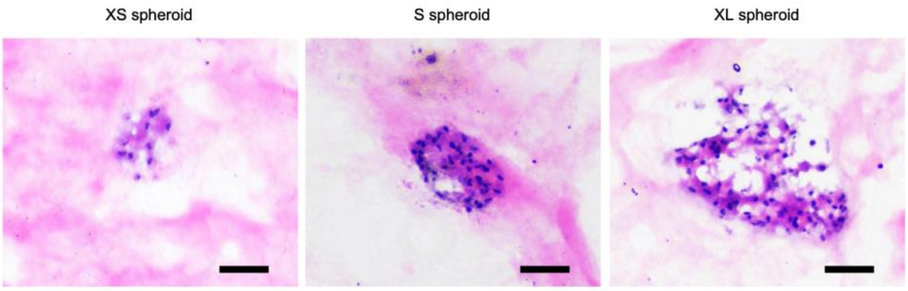

Figure S14. H&E staining of hepatic differentiation-induced hASCs as cell spheroids with different sizes in PLdECM hydrogels. Blue: cell nuclei; Red: cytoplasm; Pink: collagen. Scale bar: 50 μm.

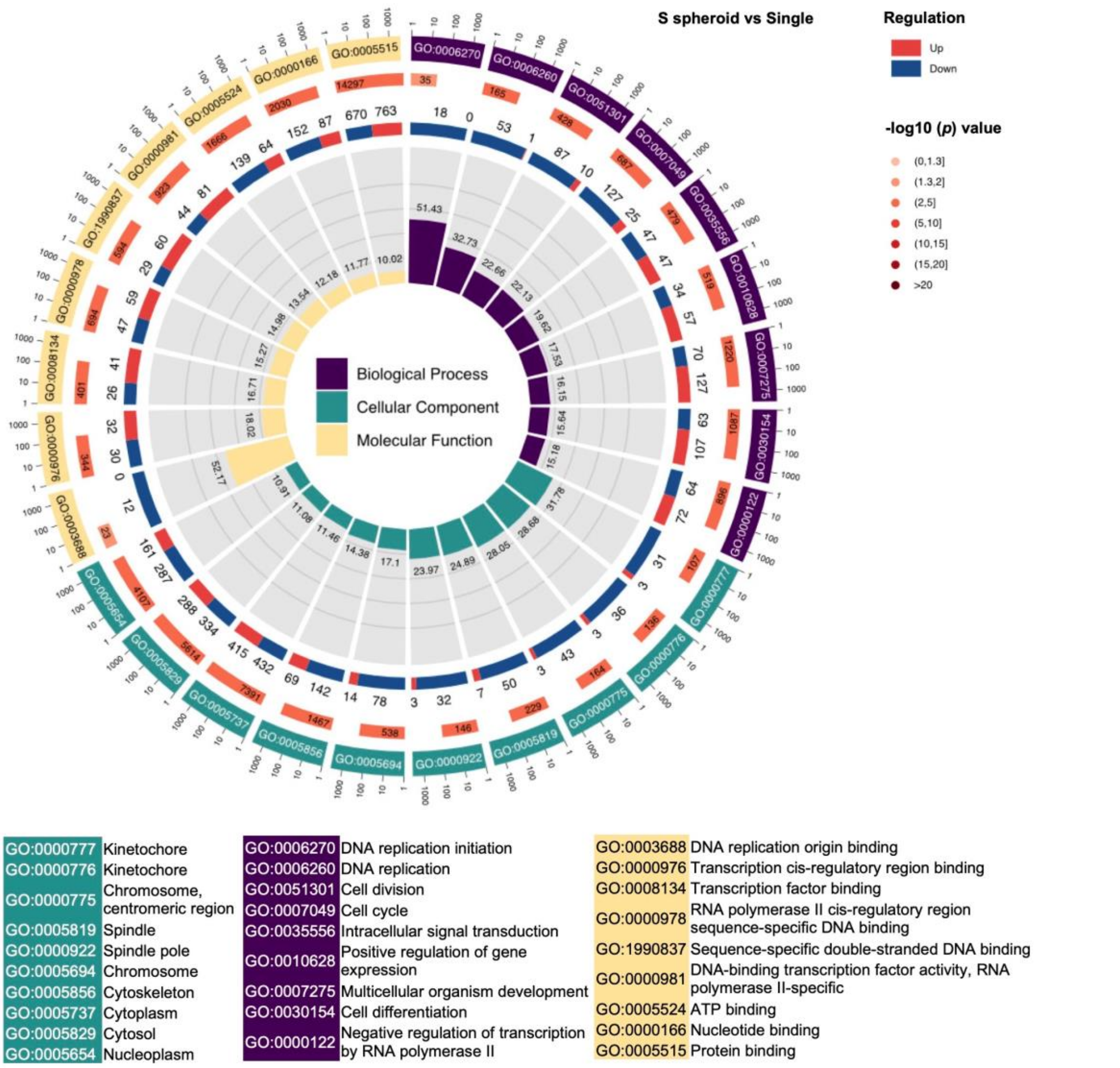

Figure S15. GO analysis of the significantly expressed genes between cell spheroids and single cells. $n = 3$, $p < 0.05$.

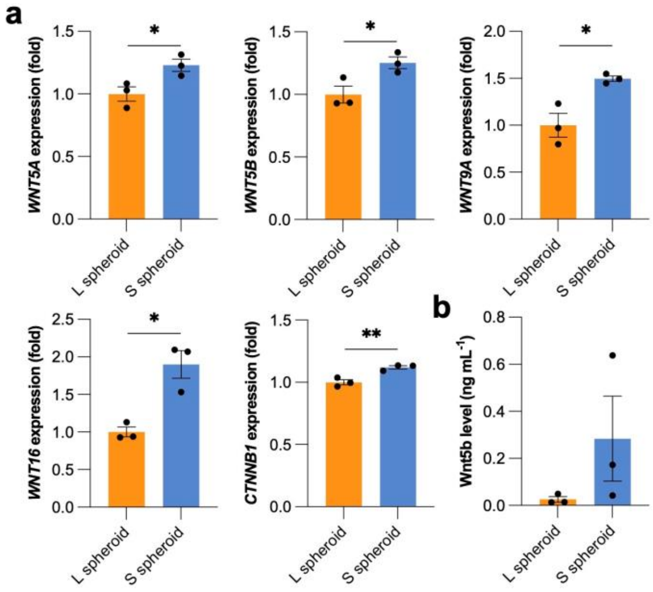

Figure S16. The characterization of Wnt signaling pathway-related molecules. (a) The relative mRNA expression levels of *WNT5A*, *WNT5B*, *WNT9A*, *WNT16*, and *CTNNB1* genes in hASC spheroids with different sizes. Data are normalized to the value of "L spheroid" group. (b) The Wnt5b level in the supernatant of hASC spheroids with different sizes. All data are presented as mean ± SEM, $n = 3$. L spheroid: cell spheroid with a diameter of 150 μm; S spheroid: cell spheroid with a diameter of 50 μm.

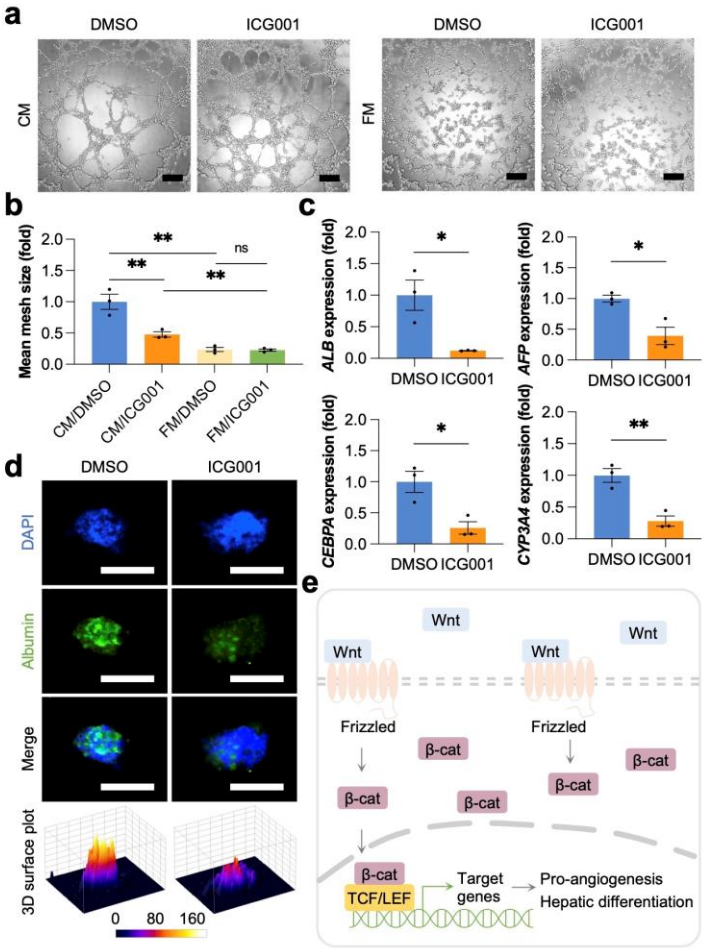

Figure S17. Wnt/β-catenin pathway mediates the enhancements of pro-angiogenic potential and hepatic differentiation in the small-sized hASC spheroids (S spheroid). (a) The optical images and (b) mean mesh size quantification of HUVECs formed tubes on the Matrigel with different medium formulae at 8 h. Scale bar: 200 μm. CM: S spheroid-derived conditioned medium; FM: fresh medium. All data are normalized to the value of "CM/DMSO" group. (c) The mRNA expression of hepatocyte-related genes in S spheroid after hepatic induction. All data are normalized to the value of "DMSO" group. (d) Immunofluorescent staining of albumin in the differentiated hASC spheroids. Green: Alexa Fluor 488-labelled albumin; Blue: DAPI-labelled cell nuclei. Scale bar: 50 μm. (e) The canonical Wnt/β-catenin signaling pathway in S spheroid. All data are presented as mean ± SEM, $n = 3$. $0.01 < {*p} < 0.05$, $0.001 < {**p} < 0.01$, $***p < 0.001$, and not significant (ns) $p > 0.05$.

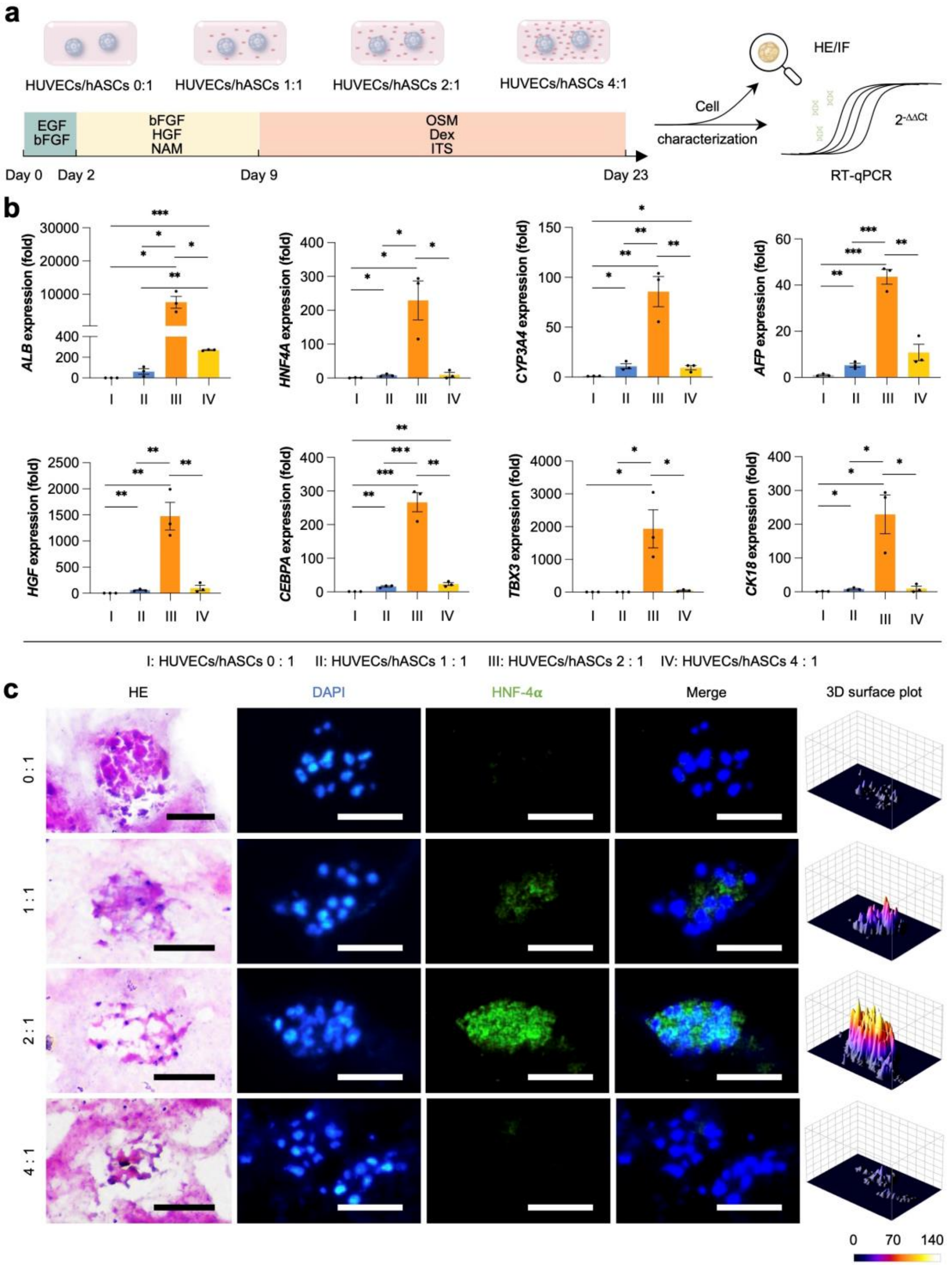

Figure S18. Co-culture of hASC spheroids with HUVECs. (a) A schematic illustrates the hepatic differentiation of small-sized hASC spheroids cultured alone or with HUVECs at a HUVECs/hASCs ratio of 1:1, 2:1, or 4:1 in PLdECM hydrogels. (b) mRNA expression of hepatocyte-related genes in hASC spheroids co-cultured with HUVECs at a HUVECs/hASCs ratio of 0:1, 1 :1, 2:1, or 4:1. Data are presented as mean ± SEM, $n = 3$, $0.01 < *p < 0.05$, $0.001 < **p < 0.01$, and $***p < 0.001$, not significant (ns) $p > 0.05$. (c) H&E staining and immunofluorescent staining of hepatic differentiation-induced hASC spheroids cultured in the PLdECM hydrogels with different ratios of HUVECs. Dark blue: cell nuclei; Red: cytoplasm;

Pink: collagen. Green: Alexa Fluor 488-labelled HNF-4α; light blue: DAPI-labelled cell nuclei. Scale bar: 50 μm.

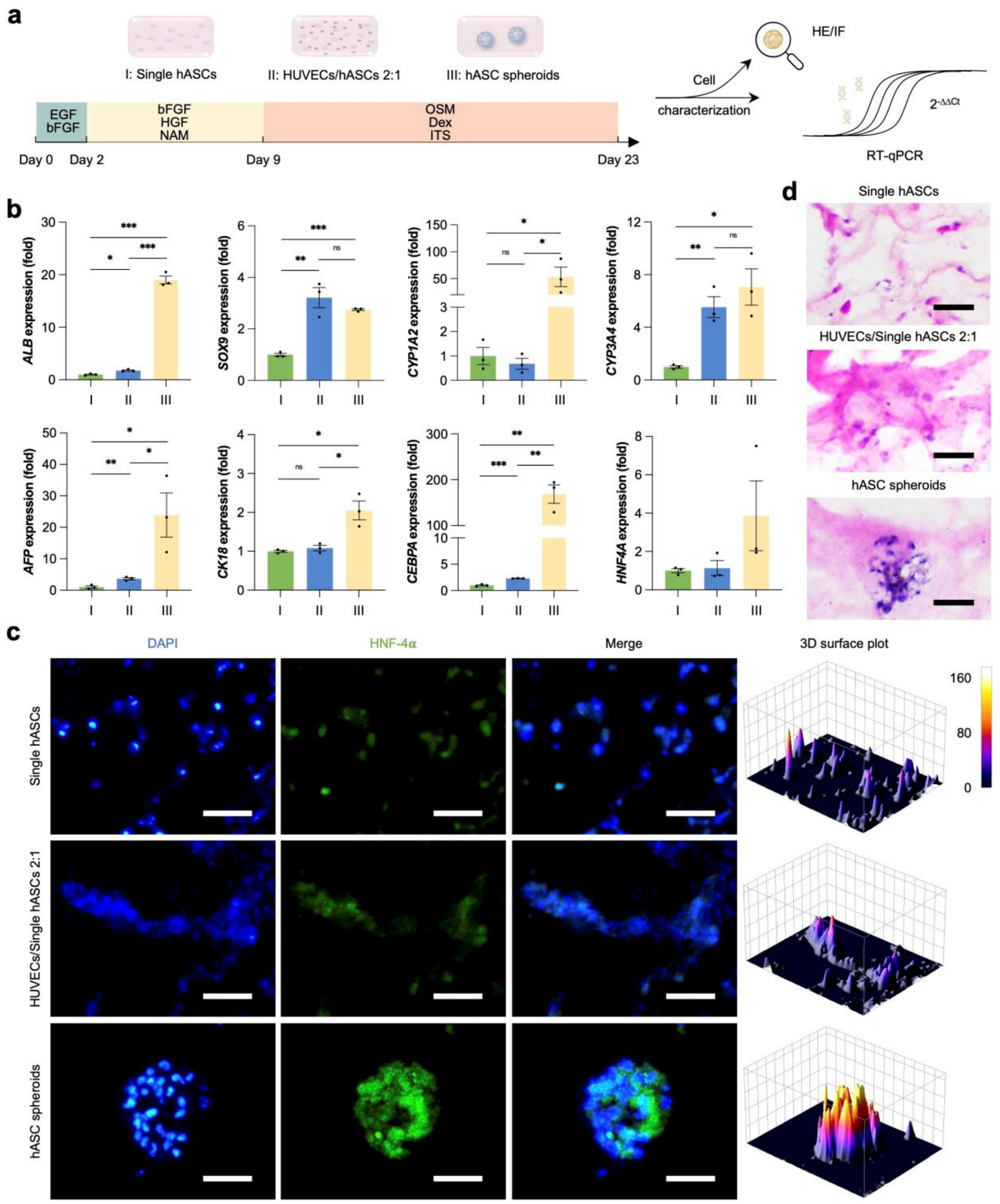

Figure S19. Hepatic differentiation of hASCs. (a) A schematic illustrates the hepatic differentiation of hASCs cultured as single cell suspension (Single hASCs) alone or cocultured with HUVECs with a ratio of 2:1 (HUVECs : hASCs), or as cell spheroids (hASC spheroids) in PLdECM hydrogels. (b) The mRNA expression of hepatocyte-related genes. All data are normalized to the value of "Single hASCs" and presented as mean ± SEM, $n = 3$, $0.01 < *p < 0.05$, $0.001 < **p < 0.01$, and $***p < 0.001$, not significant (ns) $p > 0.05$. (c) Immunofluorescent staining of HNF-4α in hepatic differentiation-induced hASCs cultured in the PLdECM hydrogels with/without HUVECs. Green: Alexa Fluor 488-labelled HNF-4α; Blue: DAPI-

labelled cell nuclei. (d) H&E staining of hepatic differentiation-induced hASCs with/without HUVECs. Dark blue: cell nuclei; Red: cytoplasm; Pink: collagen. Scale bar: 50 μm.

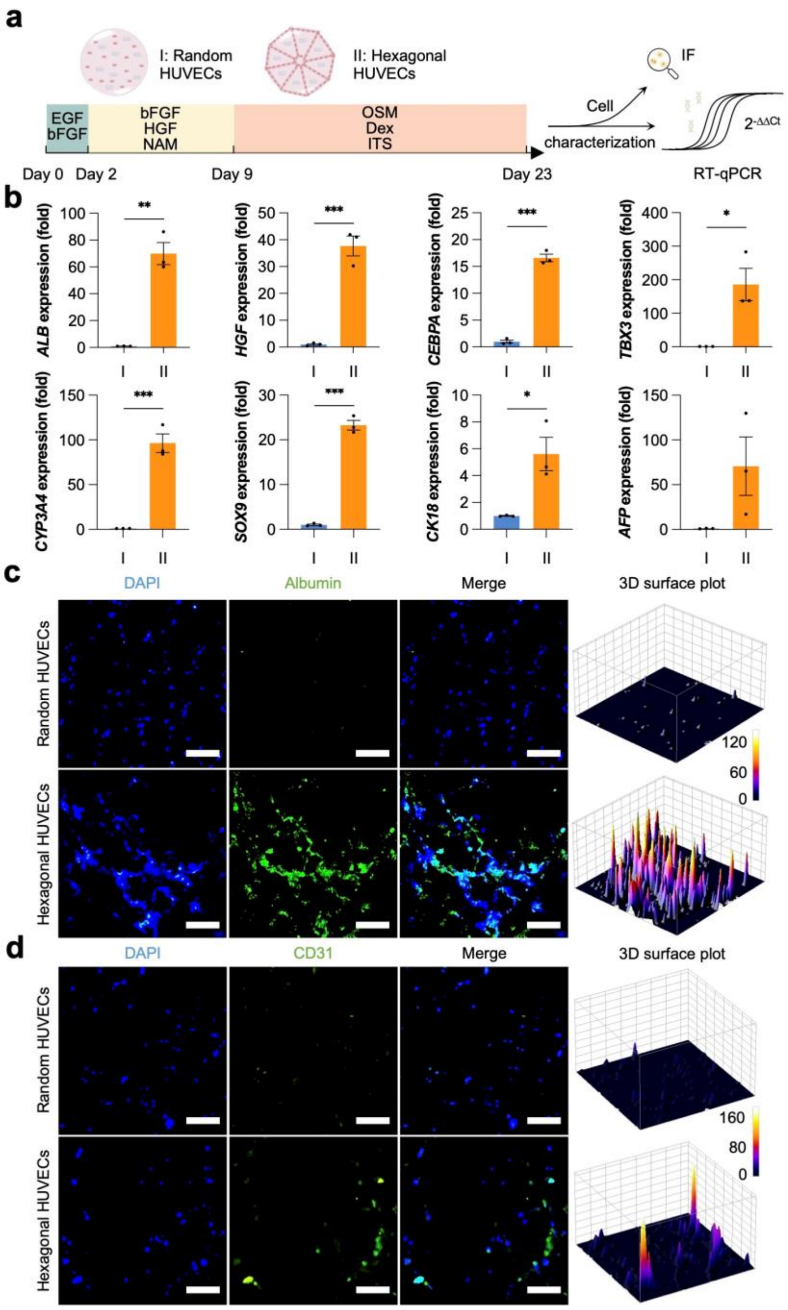

Figure S20. Co-culture of hASCs with HUVECs. (a) A schematic illustrates the hepatic differentiation of hASCs co-cultured either with randomly distributed HUVECs or hexagonally printed HUVECs. (b) The mRNA expression of hepatocyte-related genes in hASCs co-cultured with different patterned HUVECs. All data are normalized to the value of “I: Random HUVECs” group and presented as mean ± SEM, $n = 3$, $0.01 < *p < 0.05$, $0.001 < **p < 0.01$, and $***p < 0.001$, not significant (ns) $p > 0.05$. (c-d) Immunofluorescent staining images of albumin and CD31 in hASCs and co-cultured HUVECs, respectively. Blue: DAPI-labelled cell nuclei; Green: Alexa Fluor 488-labelled albumin or CD31. Scale bar: 100 μm.

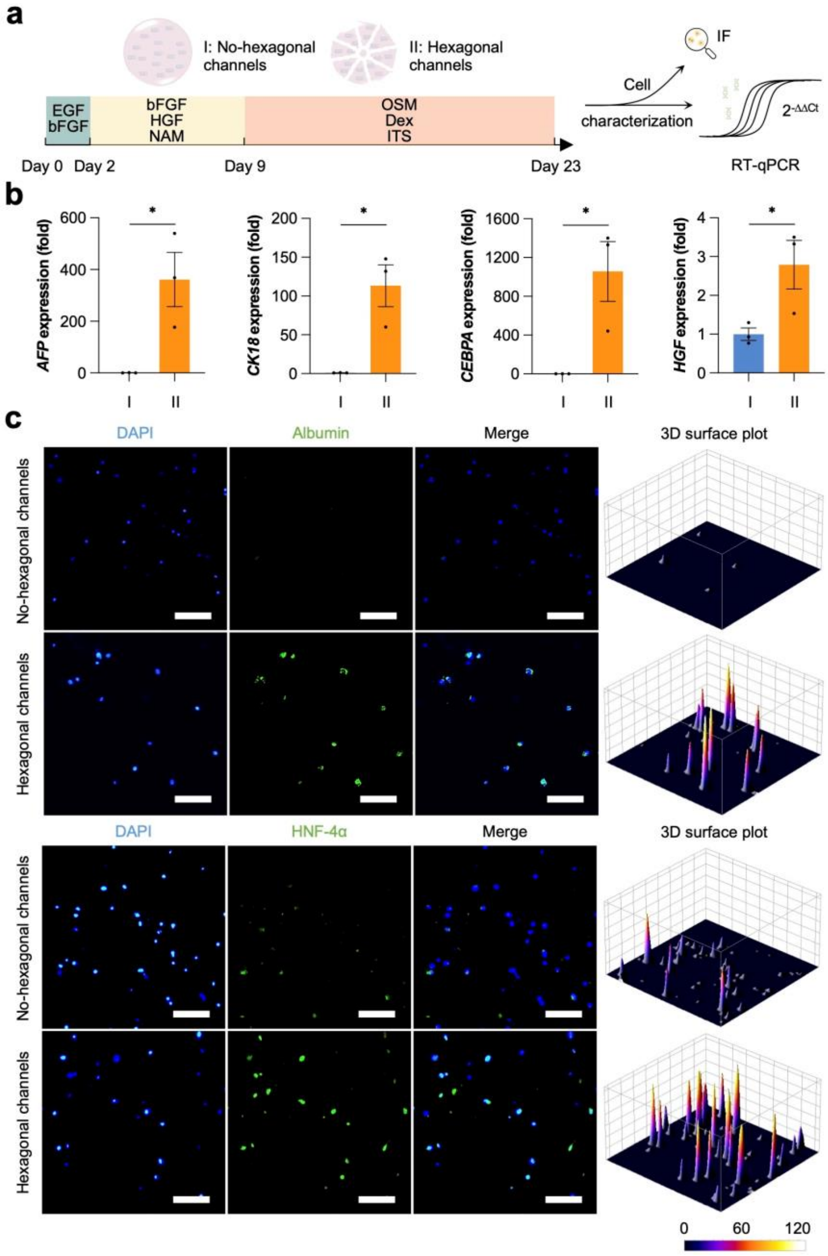

Figure S21. Hepatic differentiation of hASCs. (a) A schematic illustrates the hepatic differentiation of hASCs with/without hexagonally printed channels. (b) The mRNA expression of hepatocyte-related genes in hASCs under different conditions. All data are normalized to the value of "I: No-hexagonal channels" group and presented as mean ± SEM, $n = 3$, $0.01 < *p < 0.05$, $0.001 < **p < 0.01$, and $***p < 0.001$, not significant (ns) $p > 0.05$. (c) Immunofluorescent staining images of albumin and HNF-4α in hASCs under different conditions. Blue: DAPI-labelled cell nuclei; Green: Alexa Fluor 488-labelled albumin or HNF-4α. Scale bar: 100 μm.

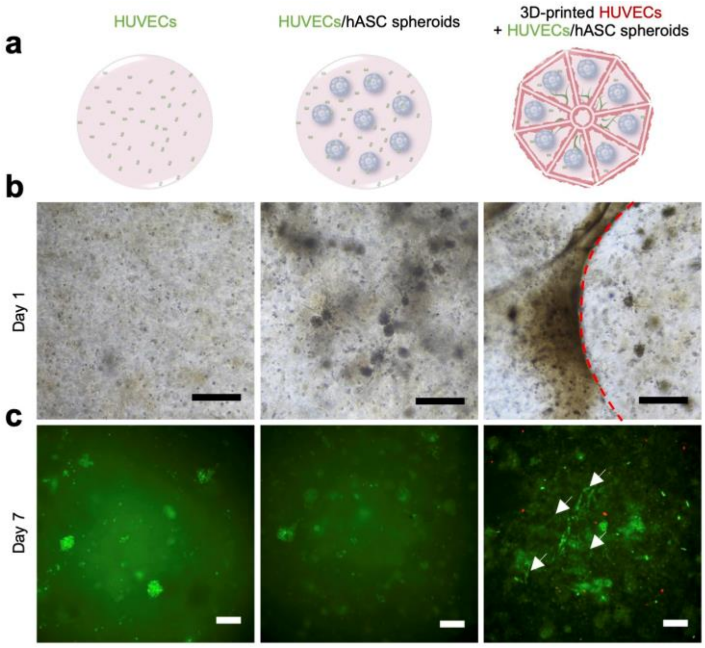

Figure S22. Interaction between hASC spheroids and HUVECs. (a) A schematic illustrates co-culture of hASC spheroids with HUVECs. (b) Optical microscopy images of HUVECs in PLdECM hydrogels, HUVECs/small-sized hASC spheroids (2:1) in PLdECM hydrogels, or co-culture with 3D-printed HUVECs on day 1. The red dash line highlights the edge of 3D-printed vasculatures. Scale bar: 500 μm. (c) Fluorescent images of HUVECs in PLdECM hydrogels, HUVECs/small-sized hASC spheroids (2 : 1) in PLdECM hydrogels, or co-culture with 3D-printed HUVECs on day 7. Green: HUVECs dispersed in PLdECM hydrogels; red: 3D-printed HUVECs. White arrows point to sprouting endothelial cells. Scale bar: 100 μm.

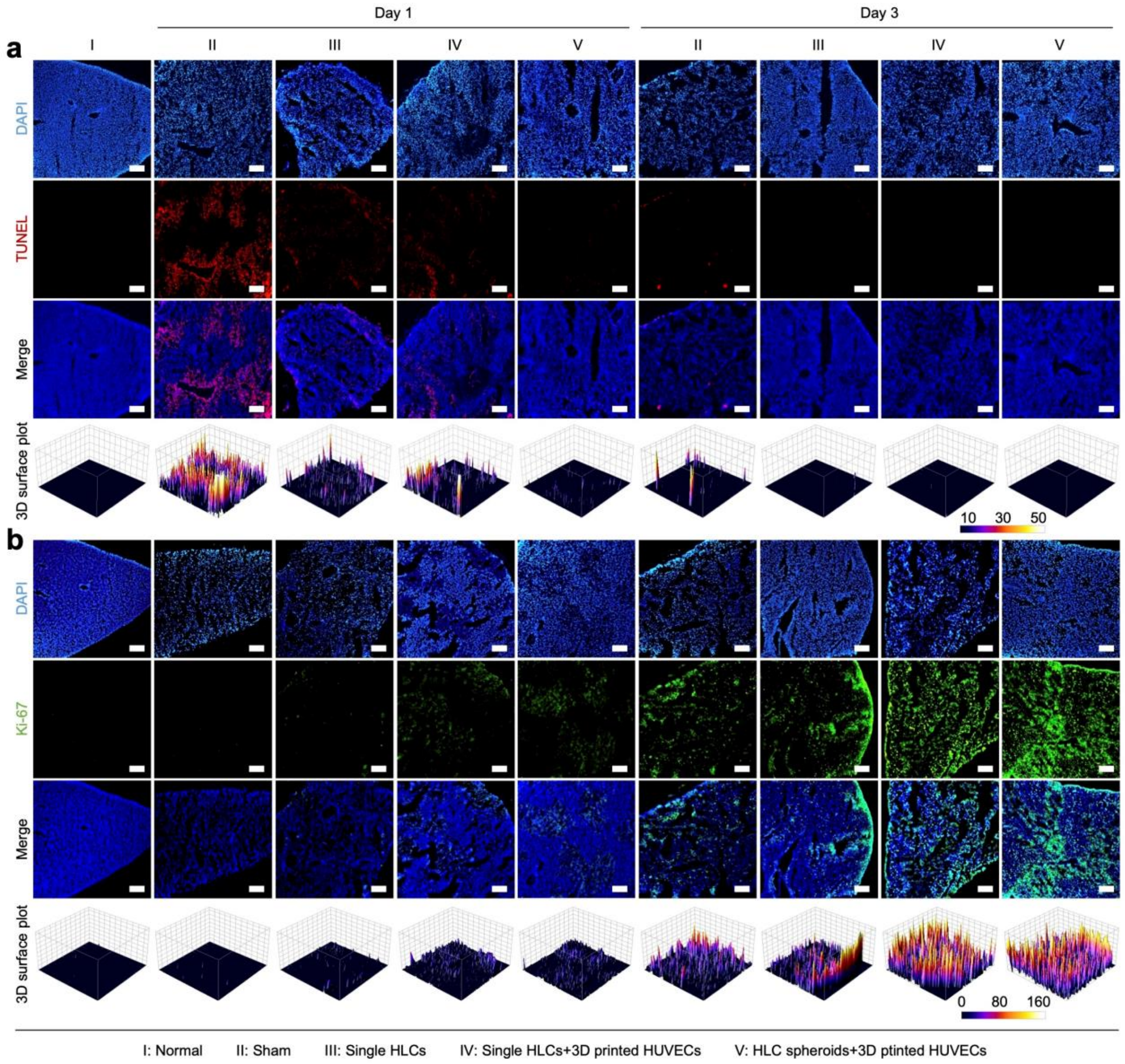

Figure S23. Immunofluorescent staining of liver tissues. (a) TUNEL staining of the mouse livers with different treatments. Red: dead cells; Blue: DAPI-labelled cell nuclei. (b) Immunofluorescent staining of Ki-67 in the mouse livers with different treatments. Green: Alexa Fluor 488-labelled Ki-67; Blue: DAPI-labelled cell nuclei. Scale bar: 200 μm. "Normal": normal mice without challenging of carbon tetrachloride (CCl_4); "Sham": CCl_4-challenged mice without treatment; "Single HLCs": CCl_4-challenged mice with subcutaneous implantation of single hASCs-derived hepatocyte-like cells (HLCs) in porcine liver-derived decellularized extracellular matrix (PLdECM) hydrogel; "Single HLCs+3D printed HUVECs": CCl_4-challenged mice with subcutaneous implantation of single HLCs in PLdECM hydrogel and 3D printed HUVECs; "HLC spheroids+3D printed HUVECs": CCl_4-challenged mice with subcutaneous implantation of HLC spheroids in PLdECM hydrogel and 3D printed HUVECs. The dosage of HLCs used in each group was kept consistent at 1×10^6 cells/mouse.

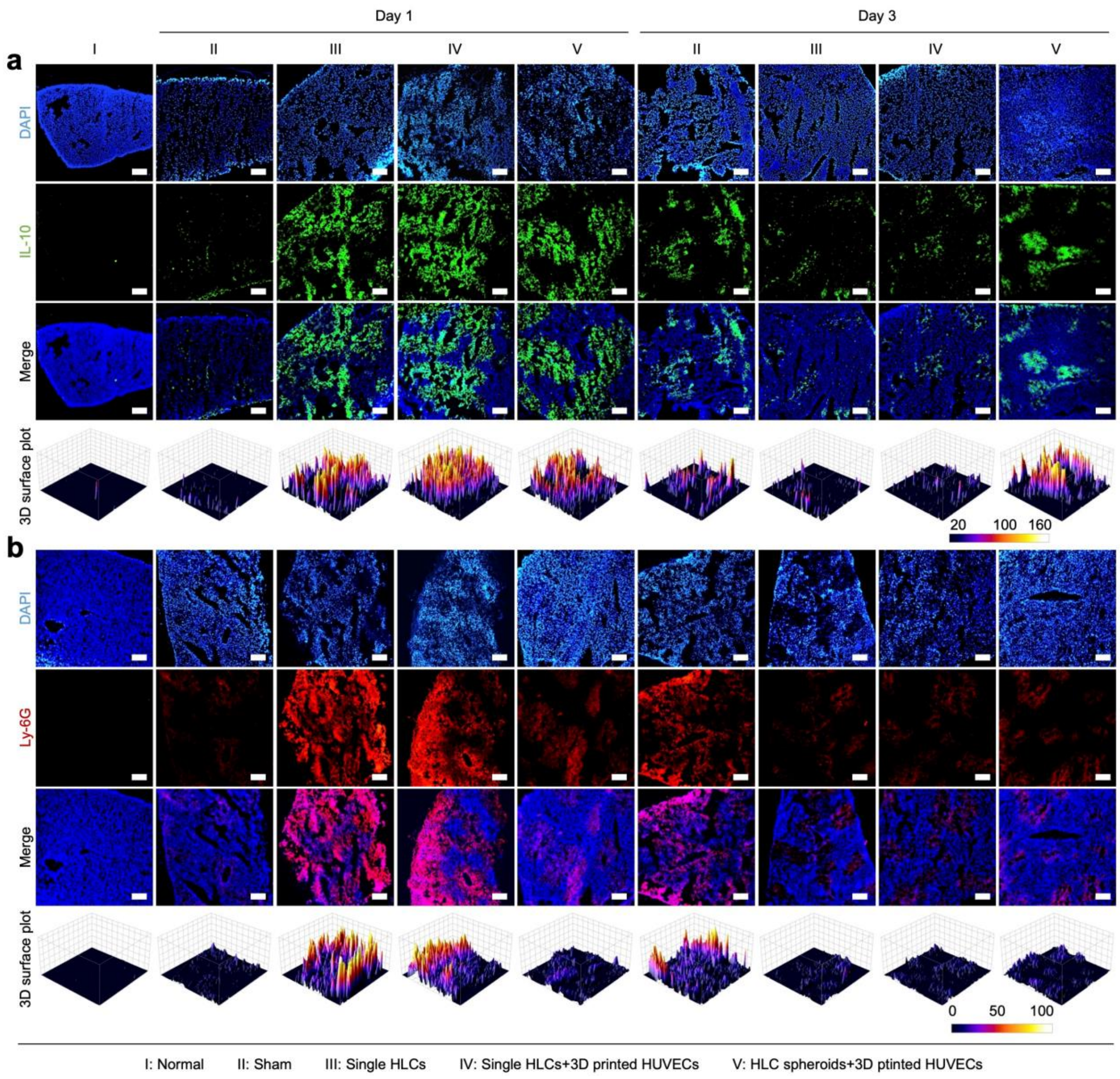

Figure S24. Immunofluorescent staining of liver tissues. (a) Immunofluorescent staining of IL-10 in the mouse livers with different treatments. Green: Alexa Fluor 488-labelled IL-10; Blue: DAPI-labelled cell nuclei. (b) Immunofluorescent staining of Ly-6G in the mouse livers with different treatments. Red: PE-labelled Ly-6G; Blue: DAPI-labelled cell nuclei. Scale bar: 200 μm. "Normal": normal mice without challenging of carbon tetrachloride (CCl_4); "Sham": CCl_4-challenged mice without treatment; "Single HLCs": CCl_4-challenged mice with subcutaneous implantation of single hASCs-derived hepatocyte-like cells (HLCs) in porcine liver-derived decellularized extracellular matrix (PLdECM) hydrogel; "Single HLCs+3D printed HUVECs": CCl_4-challenged mice with subcutaneous implantation of single HLCs in PLdECM hydrogel and 3D printed HUVECs; "HLC spheroids+3D printed HUVECs": CCl_4-challenged mice with subcutaneous implantation of HLC spheroids in PLdECM hydrogel and 3D printed HUVECs. The dosage of HLCs used in each group was kept consistent at 1×10^6 cells/mouse.

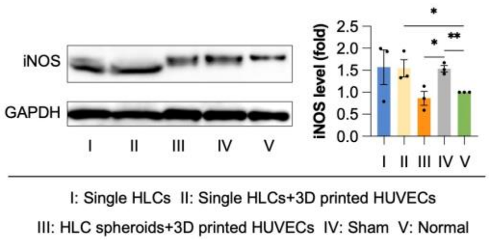

Figure S25. The western blots of GAPDH and iNOS in the mouse livers with different treatments on day 1 and their quantification by image J. GAPDH was used as the reference. All data are normalized to the value of GAPDH and presented as mean ± SEM, n = 5. 0.01 < *p < 0.05, 0.001 < **p < 0.01, and ***p < 0.001, not significant (ns) p > 0.05.

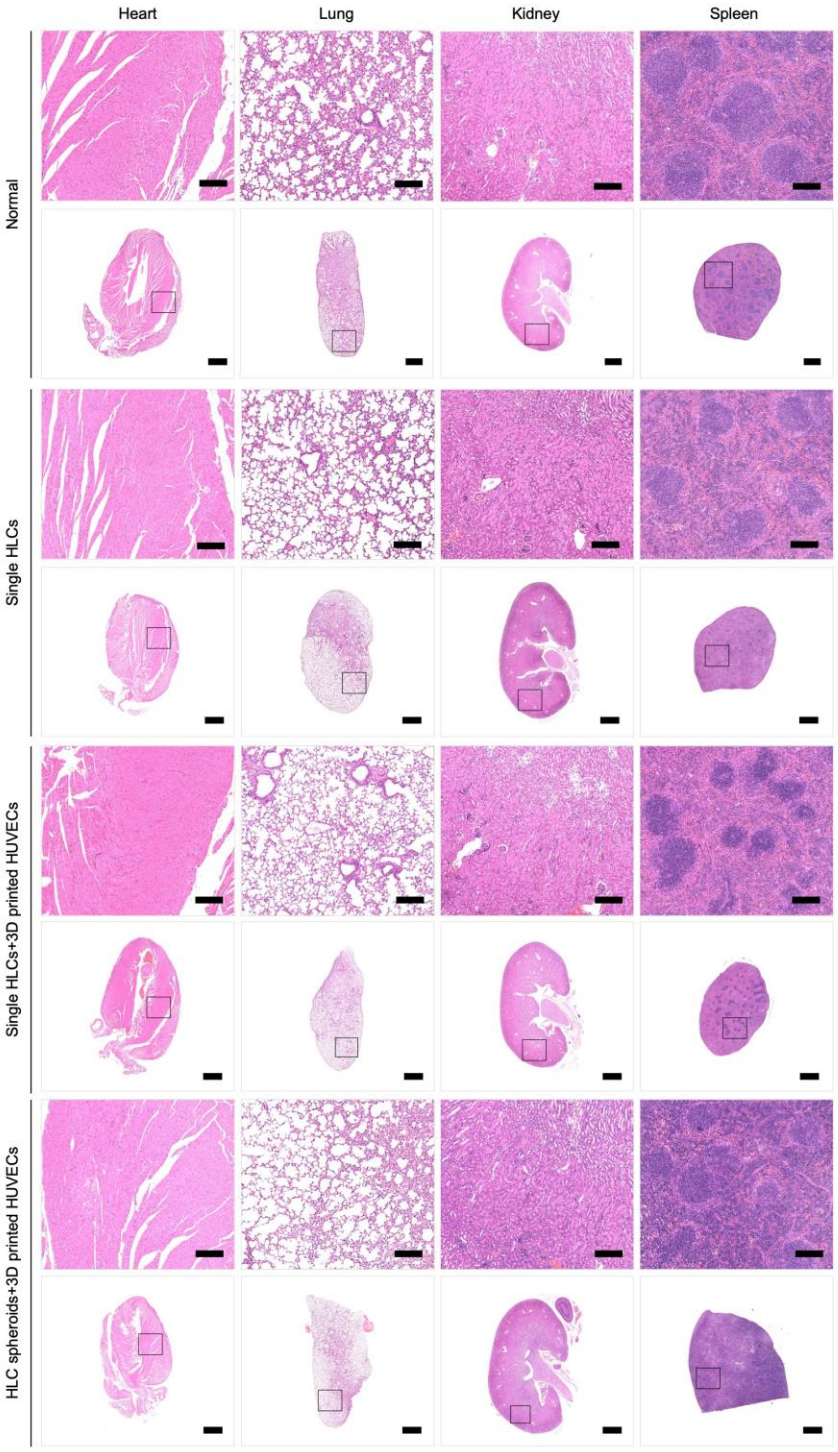

Figure S26. H&E staining of the hearts, lungs, kidneys, and spleens derived from mice with different treatments on day 3. Blue: cell nuclei; Red: cytoplasm; Pink: collagen. Scale bar: 200 μm (enlarged) and 1 cm (original). "Normal": normal mice without challenge of carbon tetrachloride (CCl_4); "Single HLCs": CCl_4-challenged mice with subcutaneous implantation of single hASCs-derived hepatocyte-like cells (HLCs) in porcine liver-derived decellularized extracellular matrix (PLdECM) hydrogel; "Single HLCs+3D printed HUVECs": CCl_4-challenged mice with subcutaneous implantation of single HLCs in PLdECM hydrogel and 3D printed HUVECs; "HLC spheroids+3D printed HUVECs": CCl_4-challenged mice with

subcutaneous implantation of HLC spheroids in PLdECM hydrogel and 3D printed HUVECs. The dosage of HLCs was kept consistent at 1×10^6 cells/mouse.

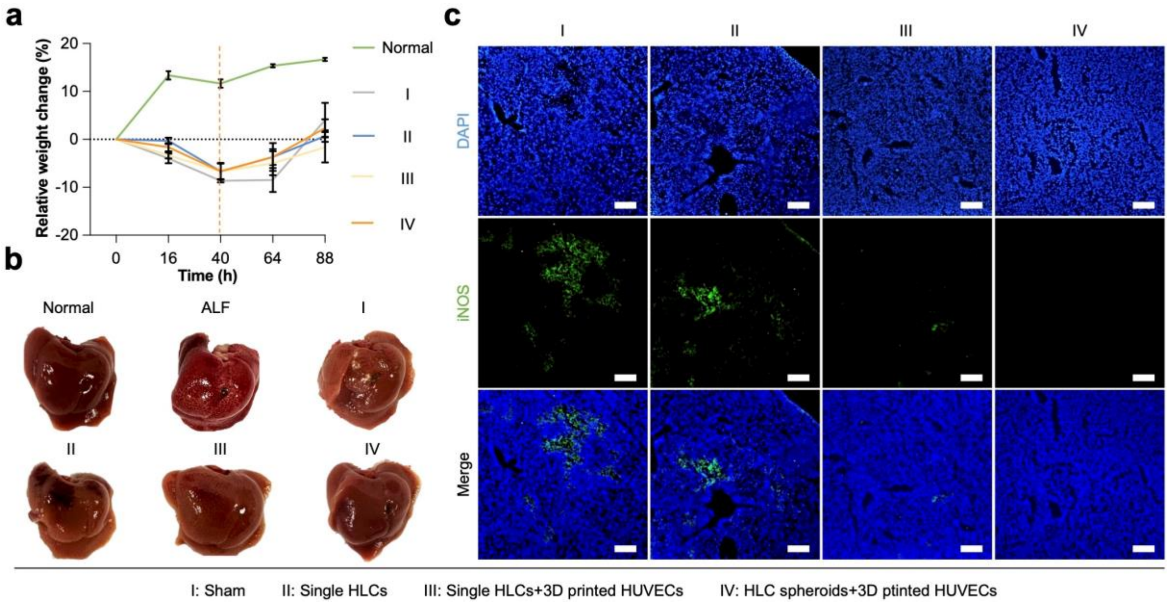

Figure S27. Characterization of APAP-challenged mice. (a) The relative weight change of Balb/c mice with different treatments. All data are normalized to the value at 0 h and presented as mean ± SEM, n = 3. (b) The gross images of mouse livers with different treatments. (c) Immunofluorescent staining of iNOS in the mouse livers with different treatments. Green: Alexa Fluor 488-labelled iNOS; Blue: DAPI-labelled cell nuclei. Scale bar: 200 μm. "Normal": normal mice without challenging of acetaminophen (APAP); "ALF": APAP-challenged mice at 16 h without treatment; "Sham": APAP-challenged mice without treatment at 88 h; "Single HLCs": APAP-challenged mice with subcutaneous implantation of single hASCs-derived hepatocyte-like cells (HLCs) in porcine liver-derived decellularized extracellular matrix (PLdECM) hydrogel at 88 h; "Single HLCs+3D printed HUVECs": APAP-challenged mice with subcutaneous implantation of single HLCs in PLdECM hydrogel and 3D printed HUVECs at 88 h; "HLC spheroids+3D printed HUVECs": APAP-challenged mice with subcutaneous implantation of HLC spheroids in PLdECM hydrogel and 3D printed HUVECs at 88 h. The dosage of HLCs was kept consistent at 1×10^6 cells/mouse.

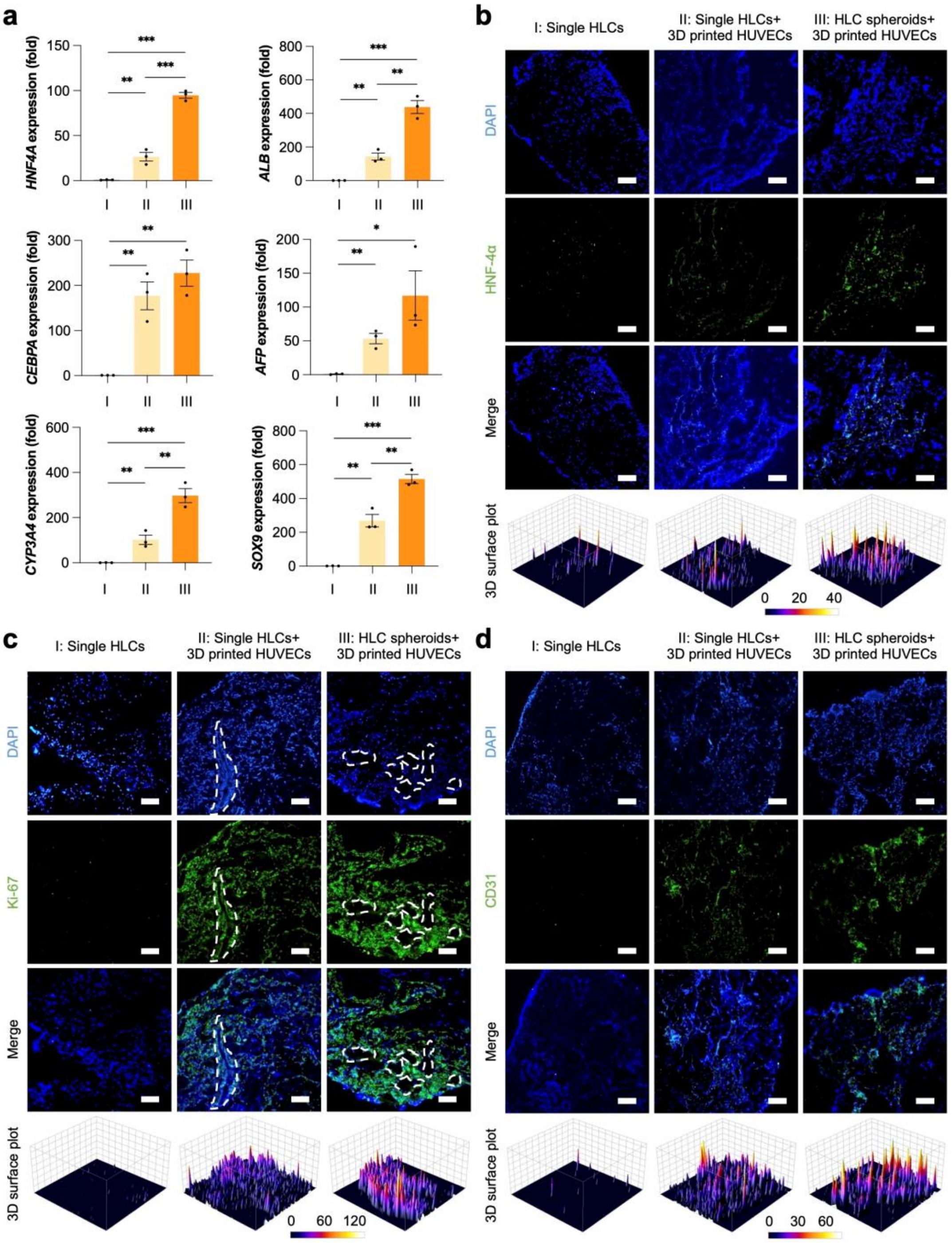

Figure S28. Characterization of the implants 3-day post-transplantation. (a) The mRNA expression of hepatocyte-related genes (*HNF4A*, *CEBPA*, *AFP*, *CYP3A4*, and *SOX9*) in the subcutaneous implants of acetaminophen (APAP)-challenged mice at 88 h. All data are normalized to the value of “I: Single HLCs” group and presented as mean ± SEM, $n = 3$, 0.01 < *p < 0.05, 0.001 < **p < 0.01, and ***p < 0.001, not significant (ns) $p > 0.05$. (b-d) Immunofluorescent staining of HNF-4α, Ki-67, and CD31 in the subcutaneous implants of acetaminophen (APAP)-challenged mice at 88 h. Green: Alexa Fluor 488-labelled HNF-4α, Ki-67, or CD31; Blue: DAPI-labelled cell nuclei. Scale bar: 200 μm. “I: Single HLCs”: APAP-challenged mice with subcutaneous implantation of single hASCs-derived hepatocyte-like cells

(HLCs) in porcine liver-derived decellularized extracellular matrix (PLdECM) hydrogel; “II: Single HLCs+3D printed HUVECs”: APAP-challenged mice with subcutaneous implantation of single HLCs in PLdECM hydrogel and 3D printed HUVECs; “III: HLC spheroids+3D printed HUVECs”: APAP-challenged mice with subcutaneous implantation of HLC spheroids in PLdECM hydrogel and 3D printed HUVECs. The dosage of HLCs was kept consistent at 1 $\times 10^6$ cells/mouse.

Table S1. The primer pairs of specific human genes.

Gene name	Primer sequences (5′→3′)
GAPDH	Forward: GTCTCCTCTGACTTCAACAGCG<br>Reverse: ACCACCCTGTTGCTGTAGCCAA
ACTB	Forward: CACCATTGGCAATGAGCGGTTC<br>Reverse: AGGTCTTTGCGGATGTCCACGT
ALB	Forward: GATGAGATGCCTGCTGACTTGC<br>Reverse: CACGACAGAGTAATCAGGATGCC
AFP	Forward: GCAGAGGAGATGTGCTGGATTG<br>Reverse: CGTGGTCAGTTTGCAGCATTCTG
HNF4A	Forward: GGTGTCCATACGCATCCTTGAC<br>Reverse: AGCCGCTTGATCTTCCCTGGAT
CYP1A2	Forward: TCATCCTGGAGACCTTCCGACA<br>Reverse: GCCACTGGTTTACGAAGACACAG
CYP3A4	Forward: CCGAGTGGATTTCCTTCAGCTG<br>Reverse: TGCTCGTGGTTTCATAGCCAGC
HGF	Forward: GAGAGTTGGGTTCTTACTGCACG<br>Reverse: CTCATCTCCTCTTCCGTGGACA
CK18	Forward: GCTGGAAGATGGCGAGGACTTT<br>Reverse: TGGTCTCAGACACCACTTTGCC
SOX9	Forward: AGGAAGCTCGCGGACCAGTAC<br>Reverse: GGTGGTCCTTCTTGTGCTGCAC
TBX3	Forward: GGACACTGGAAATGGCCGAAGA<br>Reverse: GCTGCTTGTTCACTGGAGGACT
CEBPA	Forward: AGGAGGATGAAGCCAAGCAGCT

	Reverse: AGTGCGCGATCTGGAACTGCAG
FGF2	Forward: AGCGGCTGTACTGCAAAAACGG<br>Reverse: CCTTTGATAGACACAACTCCTCTC
ANGPT1	Forward: CAACAGTGTCCTTCAGAAGCAGC<br>Reverse: CCAGCTTGATATACATCTGCACAG
CXCR4	Forward: CTCCTCTTTGTCATCACGCTTCC<br>Reverse: GGATGAGGACACTGCTGTAGAG
CXCR7	Forward: CCAAGACCACAGGCTATGACAC<br>Reverse: TGGTTGTGCTGCACGAGACTGA
ID1	Forward: GTTGGAGCTGAACTCGGAATCC<br>Reverse: ACACAAGATGCGATCGTCCGCA
MET	Forward: TGCACAGTTGGTCCTGCCATGA<br>Reverse: CAGCCATAGGACCGTATTTCGG
TGFB1	Forward: TACCTGAACCCGTGTTGCTCTC<br>Reverse: GTTGCTGAGGTATCGCCAGGAA
WLS	Forward: GTTGGCTCCTTCTGCCTCTTCA<br>Reverse: AGGCAGATTCCAGCCACGATGA
MKI67	Forward: GAAAGAGTGGCAACCTGCCTTC<br>Reverse: GCACCAAGTTTTACTACATCTGCC
CTNNB1	Forward: CACAAGCAGAGTGCTGAAGGTG<br>Reverse: GATTCCTGAGAGTCCAAAGACAG
ITGB3	Forward: CATGGATTCCAGCAATGTCCTCC<br>Reverse: TTGAGGCAGGTGGCATTGAAGG
ITGA2	Forward: TTGCGTGTGGACATCAGTCTGG<br>Reverse: GCTGGTATTTGTCGGACATCTAG

ITGAV	Forward: AGGAGAAGGTGCCTACGAAGCT<br>Reverse: GCACAGGAAAGTCTTGCTAAGGC
MMP3	Forward: CACTCACAGACCTGACTCGGTT<br>Reverse: AAGCAGGATCACAGTTGGCTGG
MMP14	Forward: CCTTGGACTGTCAGGAATGAGG<br>Reverse: TTCTCCGTGTCCATCCACTGGT
PCNA	Forward: CAAGTAATGTCGATAAAGAGGAGG<br>Reverse: GTGTCACCGTTGAAGAGAGTGG
HIF1A	Forward: TATGAGCCAGAAGAACTTTTAGGC<br>Reverse: CACCTCTTTTGGCAAGCATCCTG
VEGFA	Forward: TTGCCTTGCTGCTCTACCTCCA<br>Reverse: GATGGCAGTAGCTGCGCTGATA
TGFB3	Forward: CTAAGCGGAATGAGCAGAGGATC<br>Reverse: TCTCAACAGCCACTCACGCACA
CXCL12	Forward: CTCAACACTCCAAACTGTGCCC<br>Reverse: CTCCAGGTACTCCTGAATCCAC
IGF1	Forward: CTCTTCAGTTCGTGTGTGGAGAC<br>Reverse: CAGCCTCCTTAGATCACAGCTC

Table S2. The primer pairs of specific mouse genes.

Gene name	Primer sequences (5′→3′)
Gapdh	Forward: ATGGTGAAGGTCGGTGTGAAC<br>Reverse: GCCGTGAGTGGAGTCATACTG
Alb	Forward: AGCCCACTGTCTTAGTGAGG<br>Reverse: TCTTGCACACTTCCTGGTCC
Cyp1a2	Forward: TTCAGTCCCTCCTTACAGCC<br>Reverse: TCCAAGGCAGAATACGGTGAC
Cyp3a11	Forward: TGGTCAAACGCCTCTCCTTGCTG<br>Reverse: ACTGGGCCAAAATCCCGCCG
Hnf4a	Forward: GCTAAGGCGTGGGTAGGG<br>Reverse: AGGCTGTTGGATGAATTGAGG
Nrf2	Forward: CAGCATAGAGCAGGACATGGAG<br>Reverse: GAACAGCGGTAGTATCAGCCAG
Keap1	Forward: ATCCAGAGAGGAATGAGTGGCG<br>Reverse: TCAACTGGTCCTGCCCATCGTA
Sod1	Forward: GGTGAACCAGTTGTGTTGTCAGG<br>Reverse: ATGAGGTCCTGCACTGGTACAG
Sod2	Forward: TAACGCGCAGATCATGCAGCTG<br>Reverse: AGGCTGAAGAGCGACCTGAGTT
Gclc	Forward: ACACCTGGATGATGCCAACGAG<br>Reverse: CCTCCATTGGTCGGAACTCTAC
Nqo1	Forward: GCCGAACACAAGAAGCTGGAAG<br>Reverse: GGCAAATCCTGCTACGAGCACT
Tnfa	Forward: AGGGTCTGGGCCATAGAACT

	Reverse: CCACCACGCTCTTCTGTCTAC
Il1b	Forward: CACCTCTCAAGCAGAGCACAG
	Reverse: GGGTTCCATGGTGAAGTCAAC
Nos2	Forward: GTGCTGCCTCTGGTCTTGCAAGC
	Reverse: AGGGGCAGGCTGGGAATTCG
Il6	Forward: ACCAGAGGAAATTTTCAATAGGC
	Reverse: TGATGCACTTGCAGAAAACA
Nlrp3	Forward: AGGAGGAAGAAGAAGAGAGGA
	Reverse: AGAGACCACGGCAGAAGC
Ptgs2	Forward: AGGACTCTGCTCACGAAGGA
	Reverse: TGACATGGATTGGAACAGCA
Il10	Forward: CGGGAAGACAATAACTGCACCC
	Reverse: CGGTTAGCAGTATGTTGTCCAGC
